# Single-cell Unperturbed Activation Profiling by mass cytometry captures *in vivo* immune signaling in SLE

**DOI:** 10.3389/fimmu.2026.1844288

**Published:** 2026-08-17

**Authors:** Marie Burns, Lennard Ostendorf, Amro Abbas, Sebastian Ferrara, Heike Hirseland, Sabine Baumgart, Adrian Barreno-Sanchez, Stefan Frischbutter, Tobias Alexander, Robert Biesen, Henrik E. Mei, Andreas Grützkau, Axel R. Schulz

**Affiliations:** 1 German Rheumatology Research Center (DRFZ), An Institute of the Leibniz Association, Berlin, Germany; 2 Department of Rheumatology and Clinical Immunology, Charité – Universitätsmedizin Berlin, Freie Universität Berlin and Humboldt-Universität zu Berlin, Berlin, Germany; 3 Institute of Immunology, Core Facility Cytometry, Jena University Hospital, Friedrich-Schiller-University, Jena, Germany; 4 Institute of Allergology, Charité - Universitätsmedizin Berlin, corporate member of Freie Universität Berlin, Humboldt-Universität zu Berlin, Campus Benjamin Franklin Berlin, Berlin, Germany; 5 Fraunhofer Institute for Translational Medicine and Pharmacology ITMP, Immunology and Allergology, Berlin, Germany

**Keywords:** baricitinib, cell signaling, immune profiling, mass cytometry, precision medicine, SLE

## Abstract

**Introduction:**

Systemic lupus erythematosus (SLE) and other chronic inflammatory diseases are complex, multifactorial conditions where patient heterogeneity poses a major challenge to treatment. Despite the availability of numerous omics technologies, reliable biomarkers for precision medicine remain scarce, and many cell-based approaches are difficult to integrate into clinical settings. In this proof-of-concept study, we hypothesized that interrogating the cells’ imprinting by the inflammatory environment through Unperturbed Activation Profiling (UACT) without prior restimulation would allow for the assessment of individual immune status in chronic rheumatic diseases.

**Methods:**

We developed a highly standardized mass cytometry-based workflow to assess signaling protein phosphorylation in peripheral blood leukocytes from active SLE patients (n=20) and healthy controls (n=20) at the single-cell level. Importantly, the entire workflow from blood drawing to stabilization and preparation of barcoded samples was optimized to ensure necessary robustness of the procedure for clinical studies.

**Results:**

We identified leukocyte-specific in vivo-like signaling patterns in line with type I interferon-driven signatures, such as elevated pSTAT1 in naïve T cells and pSTAT3 in classical monocytes and naïve CD8 T cells. These patterns captured patient variability and immune activation, although they did not correlate with SLEDAI-2K disease activity scores. We further explored the utility of UACT for treatment monitoring in four SLE patients treated with the JAK inhibitor baricitinib under compassionate use, where baseline pSTAT3 levels across multiple cell subsets seemed to be associated with treatment response.

**Discussion:**

Our results thus demonstrate that single-cell UACT without restimulation of cells can detect immune activation in chronic inflammatory diseases, supporting both biomarker discovery and the identification of novel therapeutic targets.

## 1 Introduction

Chronic rheumatic diseases comprise a heterogeneous group of over 200 conditions involving inflammation, involvement of joints, connective tissue and other organs, and fluctuating activity marked by circadian and flare-remission dynamics (1–3). They develop through a complex interplay between genetic predisposition and environmental or endogenous triggers that initiate acute inflammation, which, if not properly resolved, progresses to a chronic state that ultimately causes tissue and end organ damage (4–7).

Although advances in research on pathomechanisms have enabled the development of targeted immunomodulatory therapies directed against specific cell types (e.g., B cells or plasmablasts and plasma cells) (8–12) or inflammatory mediators and their receptors (e.g., TNFα, BAFF, IL-6R, IFNAR) (13–16), routine diagnostics in rheumatology remain low-resolution and typically rely on a combination of clinical symptoms, acute-phase reactants, autoantibodies, and/or imaging (17, 18). These approaches thus do not identify which cytokines or signaling pathways drive disease in individual patients, and therefore cannot directly guide personalized treatment choices.

Heterogeneity in immunopathology, however, drives variable treatment responsiveness in patient cohorts, as seen in the non-response of a subset of rheumatoid arthritis (RA) patients to TNF blockade, or the mixed efficacy of immunosuppressive treatments in Systemic Lupus Erythematosus (SLE) (19–21). Such observations have even led to the idea that diseases like RA and SLE might actually represent groups of related conditions with distinct inflammatory drivers, rather than a single disease entity (22, 23). However, such heterogeneity is not appropriately captured by the current diagnostic tools.

Leukocyte gene expression profiling has provided insights into disease mechanisms, as well as shared signatures across diseases (e.g. type I interferon (IFN) activation), and enabled patient stratification into subtypes linked to clinical manifestations or treatment recommendations (24–27). Blood and tissue signatures have been shown to correlate well and to remain stable over time, supporting that circulating immune cells can act as sensors of the inflammatory environment in patients (27, 28). However, transcriptomic analysis, especially at the single-cell level, is expensive, and data analysis and resulting predictive gene signatures are complex, making this approach difficult to adapt for broad routine diagnostic use.

Serum cytokine measurements offer a more direct readout of inflammatory mediators and have been shown to distinguish active from inactive SLE or define immunological clusters (29, 30). Even so, cytokines are usually produced locally in inflamed tissues, their levels in peripheral blood are thus often diluted, and their detection is sensitive to sample handling, limiting utility and reproducibility (31).

Cytometry provides a rapid and robust analysis of immune cell abundance, phenotype, and function, such as cytokine production or cell signaling activity, at single-cell resolution and the protein-level. Albeit it at a smaller scale and with few parameters, cytometry is also already established in many clinical routine laboratories, making it an attractive option for the study and translation of novel clinical biomarkers. Plasmablast frequencies and SIGLEC-1 (CD169) expression on monocytes are examples of clinically useful, robust blood-derived cellular biomarkers associated with SLE activity, but they have not been established to sub-stratify patients or guide treatment choices (32–34).

More recent cytometric studies have accounted for the importance and dynamics of leukocyte activation in autoimmunity, and highlighted a role for cellular signaling and cytokine production in the pathogenesis and heterogeneity of chronic inflammatory diseases (35–40). However, none of these approaches have yielded robust biomarkers for precision medicine, and many of them have relied on stimulation-based assays that assess cellular response capacity to certain cytokines rather than native *in vivo* signaling activation states.

To overcome these limitations, we conducted a proof-of-concept study to test whether peripheral blood immune cells could be used as living biosensors of a patient’s inflammatory environment by quantifying *in vivo*-like (unstimulated) phospho-protein levels across major and minor immune cell subsets. Work in RA and in patients recovering from hip replacement surgery has demonstrated that cell signaling measurements in unstimulated whole blood can reveal disease- and recovery-associated alterations, supporting the technical viability of this approach in inflammatory disease contexts (41, 42). Our mass cytometry-based approach, termed Unperturbed Activation Profiling (UACT), employs a highly standardized fixation-based sample collection protocol to interrogate native immune cell signaling activity without restimulation. By focusing on pathway activity rather than cell composition or response potential after stimulation, we aimed to capture the *in vivo*-like signaling state of a cohort of 20 SLE patients and matched healthy controls as accurately as possible.

SLE serves as a useful test case for developing cell-signaling based biomarkers. It is a chronic, multifactorial autoimmune disease marked by autoantibodies to nuclear antigens, prominent type I IFN involvement, and broad activation of both the innate and adaptive immune system (23, 43, 44). Clinical manifestations are very heterogeneous, ranging from nephrological and dermatological to musculoskeletal, cardiorespiratory, and neuropsychiatric symptoms, that evolve dynamically and appear in varying combinations (17). This heterogeneity complicates diagnosis and treatment, underscoring the need for early, precise, and individualized biomarkers.

Here, we show that active SLE is characterized by selective induction of pSTAT1, pSTAT3, and pSTAT5 in specific cell types and differentiation states in this cohort. UACT enables the definition of exploratory patient subgroups based on distinct phosphorylation patterns that complement, but are not redundant with, conventional measures of disease or IFN activity, or autoantibody profiles.

Thus, our proof-of concept study supports UACT as a highly standardizable, and scalable approach to capture patient heterogeneity to ultimately advance biomarker-guided precision medicine in SLE and other chronic inflammatory diseases.

## 2 Materials and Methods

### 2.1 Patient and healthy control samples

Blood samples were obtained from SLE patients recruited from the outpatient clinic of the Charité-Universitätsmedizin Berlin, Department of Rheumatology and Clinical Immunology, and from healthy controls. Patients were diagnosed according to the 2019 EULAR/ACR classification criteria (17). Clinical manifestations, SLEDAI-2K and/or BILAG, and relevant prescription medication were recorded by the treating physicians. Autoantibody profiles and titers, leukocyte counts, complement protein levels and monocytic SIGLEC-1 expression were obtained from Labor Berlin GmbH as part of the clinical routine, and recorded by treating physicians. Patient characteristics are summarized in **Table 1** and ST2, ST3.

**Table 1:** Main characteristics of the cohort (for more details, please see **Supplementary Materials**, tables ST2 and ST3).

Characteristics	Sex, medication	Controls	SLE		
group size (n)		20	20		
sex	f	18	18		
	m	2	2		
age (median, IQR)		39 (30-46)	39 (30-47)		
SLEDAI-2k (median, range)		n.a.	8 (2-14)		
immunosuppressive medication		n	n	median dose	range
	prednisone (mg/d)	n.a.	15	5	0-20
	hydroxychloroquine (mg/d)	n.a.	17	300	0-400
	methotrexate (mg/w)	n.a.	3	15	0-15
	azathioprine (mg/d)	n.a.	4	100	0-100
	Belimumab (mg/w)	n.a.	3	200	0-800

Four SLE patients undergoing compassionate use treatment with the Janus kinase inhibitor baricitinib (4 mg/day, oral administration) were recruited from the outpatient clinic of the Charité Universitätsmedizin Berlin, Department of Rheumatology and Clinical Immunology. Blood samples were obtained before treatment, and one, two, four, eight and twelve weeks after starting.

The study of blood samples was conducted in accordance with the guidelines of the Declaration of Helsinki and approved by the Ethics Committee of the Charité-Universitätsmedizin Berlin (EA1/132/16). Informed consent was obtained from all subjects. Written informed consent was obtained from the individual for the publication of any potentially identifiable images or data included in this article.

Within 30 minutes after phlebotomy, heparinized whole blood samples were cryopreserved using Proteomic Stabilizer (Smart Tube Inc., Las Vegas, NV, USA) (**Supplementary Figure 1**). 500 µL of whole blood were mixed with 700 µL of Proteomic Stabilizer-1, incubated at room temperature for 12 min and then transferred to a -80 °C freezer. Samples were stored at -80 °C until analysis. Serum collection tubes were stored at room temperature for 30 minutes after phlebotomy and subsequently centrifuged at 1000 x g for 10 minutes. Serum samples were stored at -80 °C until further processing.

### 2.2 Analysis of peripheral blood leukocytes by mass cytometry

**Figure 1:**
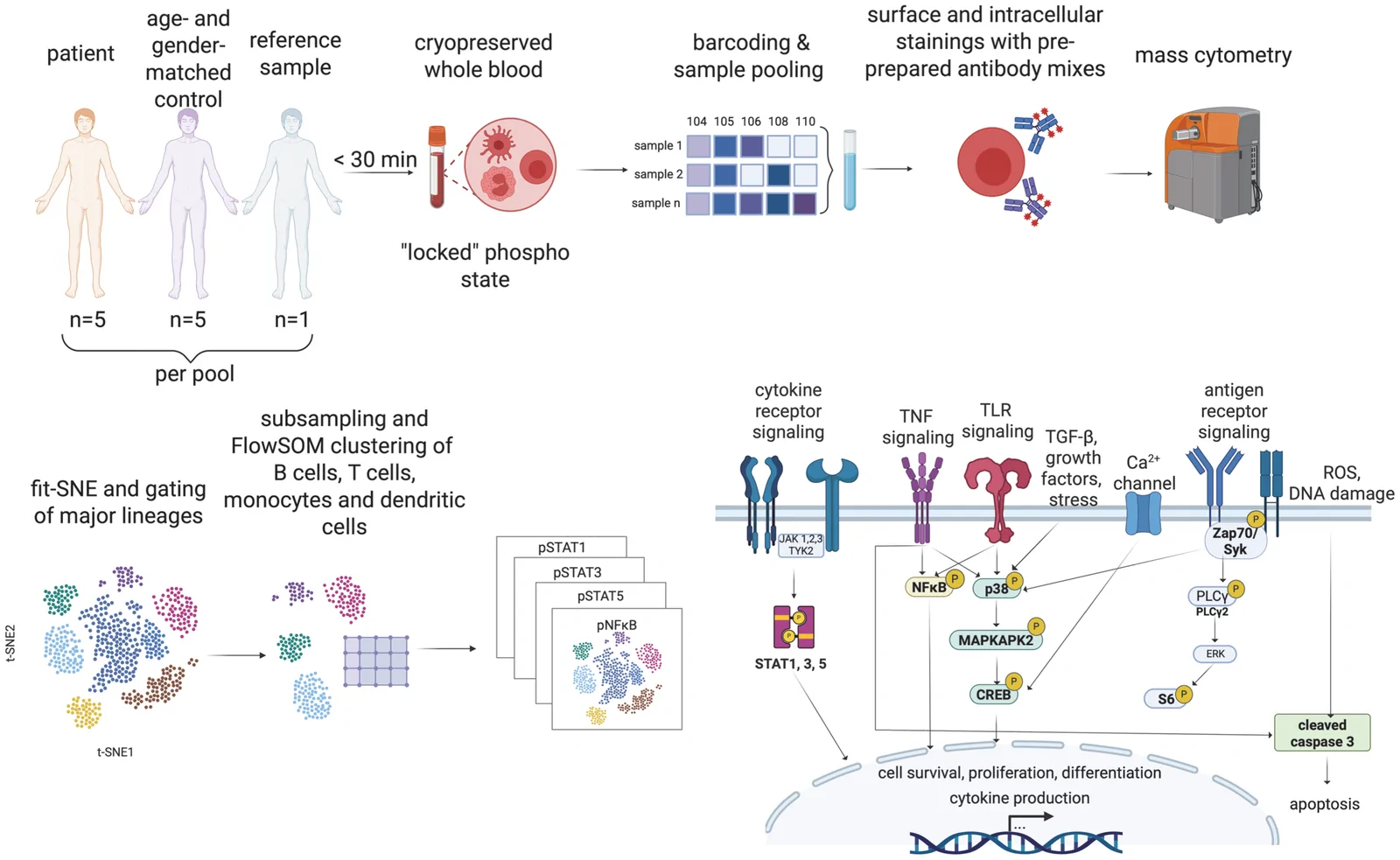
A fixative-based whole blood analysis pipeline to assess cell-type specific UACT leukocyte signaling pathway activation. On a given measurement day, five patient and five matched control samples, as well as the reference sample, were subjected to sample barcoding, cell-surface and intracellular antibody stainings with pre-prepared frozen antibody cocktails, before acquisition on the mass cytometer (top). After data pre-processing, leukocytes were subjected to dimension reduction and clustering to identify major and minor subsets and interrogate the phosphorylation states of signal transduction molecules involved in cytokine, antigen and Toll-like receptor signaling shown in (bottom). Created in BioRender. Burns, M. (2026) https://BioRender.com/c67f4l1.

Each mass cytometry experiment comprised five randomized, paired samples from SLE patients and five age- and sex-matched control samples, as well as one healthy control sample serving as technical reference to monitor assay performance (**Figure 1**). Samples were thawed in a water bath at approx. 10 °C, transferred into a 50 mL centrifuge tube containing 5 mL of 1x Thaw-Lyse buffer (Smart Tube Inc.) and incubated at room temperature for 10 min to lyse erythrocytes. Cells were then centrifuged at 700 x g for 5 min, before repeating the lysis step with 25 mL of 1x Thaw-Lyse buffer for 5 min at room temperature. After completing the erythrocyte lysis, the samples were washed twice with cell staining medium (CSM, 1x PBS (10x stock solution obtained from Rockland Immunochemicals, Gilbertsville, PA, USA) supplemented with 0.5% (w/v) bovine serum albumin (PAN Biotech, Aidenbach, Germany) and 0.02% sodium azide (Sigma Aldrich, St. Louis, MO, USA)). Cell counts were determined for each sample by volumetric measurement using a MACSQuant 10 Analyzer (Miltenyi Biotec, Bergisch-Gladbach, Germany). Cell counts were adjusted to 1.6 x 10^6^ cells per sample for further processing.

For each run, ten study samples were barcoded using isothiocyanobenzyl (ITCB)-EDTA comprising complexed isotopically enriched palladium isotopes (atomic masses 104, 105, 106, 108 and 110) (45) in a five-choose-two-scheme for 20 min at room temperature in PBS at previously optimized concentrations. The reference sample was labeled with rhodium103-maleimide- dodecane tetraacetic acid (Rh-mDOTA). After incubation, cells were washed twice with 3 mL CSM and then pooled for further processing. For Fc receptor blocking, cells were incubated with beriglobin (CSL Behring, King of Prussia, PA, USA) for 10 minutes at room temperature), before washing cells once with 2 mL CSM. Next, cells were incubated for 15 min at room temperature in CSM supplemented with 100 U/mL sodium heparin to further minimize nonspecific reagent binding (46). Cell surface staining (47) was performed in CSM in the presence of 50 U/mL heparin for 30 minutes at room temperature. Antibodies used for cell-surface staining are listed in **Supplementary Table 1**. Antibody cocktails for surface and intracellular stainings were pre-prepared, aliquoted for each study day, and stored at -80 °C until use to minimize error and assay variability (48). The incubation was stopped by adding 2 mL CSM, and, after pelleting, cells were washed once with 2 mL PBS, and then fixed in 4% paraformaldehyde solution in PBS for 10 min at room temperature. The pooled sample was centrifuged at 700 x g for 5 min, and cells were resuspended in 1.6 mL -20 °C ice-cold pure methanol (-20 °C, Carl Roth, Karlsruhe, Germany) and transferred to 1.6 mL cryovials (Sarstedt Inc., Nümbrecht, Germany) for overnight storage at -80 °C. On the day of acquisition, the sample was washed twice with 4 mL CSM, followed by 15-minute incubation in CSM/heparin at room temperature. Antibodies for intracellular staining (**Supplementary Table 1**) were added to the heparinized cell suspension and incubated at room temperature for 60 minutes, with gentle mixing after approximately 30 minutes to counteract sedimentation. Next, the sample was washed once with 2 mL CSM and once with 2 mL PBS, followed by another fixation using 2% paraformaldehyde in PBS for 10 min at room temperature. Cells were then pelleted and resuspended in 2 mL PBS containing 1:500 (v/v) 0.125 mM iridium intercalator (Fluidigm, South San Francisco, CA, USA) for 25 minutes at room temperature. Finally, cells were washed twice with 2 mL cell staining medium, and once with 2 mL de-ionized water, before filtering through a 35 µm cell strainer cap (Corning, Corning, NY, USA) and volumetrically determining the cell count using a MACSQuant Analyzer 10. The cell concentration of the pooled sample was adjusted to 0.75 x 10^6^ cells/mL in de-ionized water, and the sample was supplemented with 10% (v/v) EQ Four Element Calibration Beads (Standard BioTools, South San Francisco, CA, USA). Cells were acquired on a Helios mass cytometer (Standard BioTools) at a rate of up to 350 events per second, using a lower convolution threshold of 400, a minimum event duration of 10, a maximum event duration of 150, a sigma value of 3, and noise reduction and randomization switched on.

### 2.3 Data analysis

#### 2.3.1 Pre-processing

Raw mass cytometry data were converted to Flow Cytometry Standard (FCS) 3.0 files during acquisition on the Helios mass cytometer. Data files of each sample pool were normalized based on signals of EQ Four Element Calibration Beads spiked into the sample (passport P13H2302) using the Helios software version 6.7.1014 (Standard BioTools). Next, all cytometric channels except time, event length and Gaussian parameters were subjected to arcsinh transformation with a scale argument of 5. Data of beads were excluded based on their ^140^Ce and ^151^Eu signals using manual gating in FlowJo version 10.8.2 (BD Biosciences, Franklin Lakes, NJ, USA). Intact cells were identified according to DNA staining with iridium intercalator, and events with an event length value greater than 30 were excluded to minimize the presence of doublet events in the dataset. A cleaved caspase 3-specific antibody was included in the antibody panel to identify apoptotic cells. However, in line with the short time between phlebotomy and blood sample fixation, we observed only few cleaved caspase 3-positive cells, mostly monocytes, without significant differences detectable between SLE samples and controls. Finally, leukocytes were gated according to their CD45 expression. Data of individual samples were extracted by Boolean gating based on barcode signals using manual gating in FlowJo version 10.8.2 (BD Biosciences, Franklin Lakes, NJ, USA) (49). Compensation of signal spillover was performed in R Studio (version 1.2.1335) using CATALYST (version 1.8.7) (50, 51). Percentile-based batch normalization (95^th^ percentile for surface markers, 99^th^ percentile for intracellular markers; see marker overview in **Supplementary Table 1**) of the sample pools acquired on different study days was performed using CytofBatchAdjust (https://github.com/CUHIMSR/CytofBatchAdjust) in R Studio (version 1.2.1335) based on the reference samples included in each sample pool, as previously described (52). Batch normalization did not significantly impact the detection of cell frequencies, surface markers and phospho-STAT levels in anchor samples, or group differences between SLE patients and healthy controls (see **Supplementary Figures 2**, **3**).

#### 2.3.2 Dimension reduction and clustering

Debarcoded, compensated, batch-normalized data were uploaded to omiq.ai (Dotmatics, Santa Clara, CA, USA) and downsampled to 100.000 cells per sample. Dimension reduction was performed using the fi-tSNE (53) implementation with an early exaggeration rate of 250, a maximum number of 750 iterations and a learning rate of total cell number divided by 36. The markers used for dimension reduction are listed in **Supplementary Table 1**. Next, islands corresponding to major leukocyte subsets were identified in the fi-tSNE plot based on lineage marker expression profiles (**Supplementary Figure 4**). Neutrophils, basophils, eosinophils were gated from this plot. Total NK (natural killer) cells were gated from the fi-tSNE and then manually gated to identify CD16+CD56+ NK cells and CD16+/- CD56high NK cell subsets. B cells and plasmablasts/plasma cells (PB/PC), T cells, monocytes and dendritic cells were subjected to FlowSOM clustering using a 10 x 10 grid, Euclidean distance, and consensus metaclustering. Markers used for cell type-specific dimension reduction and FlowSOM clustering are listed in **Supplementary Table 1**. The number of metaclusters was set to 15 for B cells including plasmablasts/plasma cells, to 15 for T cells, and to 5 for monocytes and dendritic cells (**Supplementary Figure 4**). For T cells, two overlapping naïve T cell and two CD4 central memory T cell clusters were re-merged, leading to 13 total T cell clusters. Similarly, for B cells, four naïve and two IgG+ and IgM+ clusters, respectively, were recombined, yielding 10 final B cell clusters. One cluster in the monocyte and DC (dendritic cell) clustering showed inconclusive lineage marker expression and was excluded from further analyses (**Supplementary Figure 5**). Overall, the analysis of UACT signals in healthy controls and patients thus included 32 cell subsets.

#### 2.3.3 Statistical analysis

Comparisons between healthy controls and SLE patients, as well as between clinical subgroups of patients, were performed using the statistical tests indicated in the corresponding figure legends. For analyses involving ANOVA with multiple comparisons, p-values were adjusted using either Tukey’s or Šidák’s correction, as appropriate. Correlations between cytometric data and clinical parameters, such as SLEDAI-2K, complement or autoantibody levels, were analyzed using Spearman correlation in GraphPad Prism version 9.3.1 (Dotmatics), which yielded unadjusted p-values. For correlation analyses of cytometric data with serum cytokine levels, R packages psych (version 2.2.5, function corr.test), stats (version 4.2.1, function p-adjust), boot (version 1.3.28, functions boot, boot.ci) and corrplot (version 0.92, function corrplot) were used. Spearman’s rank correlation coefficients were calculated and the statistical significance was confirmed using equi-tailed two-sided nonparametric confidence intervals based on 1000 bootstrap samplings. The plots show non-adjusted significant correlation coefficients (p < 0.05) that were used for further analysis, and should be considered exploratory.

#### 2.3.4 Volcano plots

To identify differences in cell abundances and signal intensity data between SLE and control samples, cluster cell counts and arcsinh (5)-transformed mean marker signal intensities were exported from omiq.ai and analyzed using GLMM and LMM in diffcyt (54, 55). For determining differences in signal intensities, we only considered clusters containing at least five cells in more than half of the samples in each sample group. Multiple testing correction was performed using the Benjamini-Hochberg method. Log2 fold changes and log10 p-values were calculated and volcano plots were generated using the enhanced volcano package (version 1.13.2). R version 4.1.1. and packages readxl (version 1.3.1), diffcyt (version 1.12.0), lme4 (version 1.1.-27.1), multcomp (version 1.4-18), stats (version 4.1.1), openxlsx (version 4.2.5), gtools (version 3.9.2) were used.

#### 2.3.5 Multidimensional scaling

Multidimensional scaling (mds) was performed in R Studio (version 1.2.1335) using the stats (version 3.6.1), tidyverse (version 1.3.1), and ggplot2 (version 3.3.5) packages. Cluster frequencies or arcsinh (5)-transformed signal intensity data were imported and processed using the function cmdscale from the R stats package. For plotting combined abundance and phosphorylation data, values were z-transformed before performing mds.

#### 2.3.6 Heatmaps

The clustered heatmaps showing the per-protein or per-patient phosphoprotein signal intensities were generated using the pheatmap package (version 1.0.12) in R Studio (version 1.2.1335). Clustered heatmap including all healthy donors and SLE patients was generated using the ComplexHeatmap package (version 2.0.0). For this clustering, we used the 15 UACT features identified as most significantly altered between SLE patients and healthy controls. Because these features were selected based on their group-level significance in the same dataset, this clustering approach is inherently exploratory.

### 2.4 Serum cytokine measurements

Serum cytokines were quantified using a bead-based multiplex cytokine assay (Cytokine/Chemokine/Growth Factor 45-Plex Human ProcartaPlex Panel 1, Thermo Fisher Scientific). Prior to assay protocol, all serum samples were diluted 1:3 v/v in dilution buffer provided with the kit. The samples were incubated with antibody coated magnetic beads for 30 min at room temperature with shaking, then incubated overnight at 4 °C followed by a 1 h incubation at room temperature on the next day. All subsequent incubation steps were performed according to the manufacturer’s instructions. The assay plates were read using a Luminex MAGPIX system and quantified using the xPONENT analysis software (Luminex Corp, Austin TX, USA).

## 3 Results

### 3.1 A novel fixative-based blood analysis pipeline to assess cell-type specific leukocyte signaling pathway activation

To perform cell type-, cell state- and disease-specific Unperturbed Activation Profiling (UACT) across major peripheral blood immune cell lineages and evaluate its utility for patient stratification, whole blood samples were fixed and cryopreserved within 30 minutes after phlebotomy to accurately capture degradation-sensitive native protein phosphorylation states (**Figure 1**). Timely fixation of samples was important to prevent time-dependent decline of phospho-STAT (pSTAT) signals (**Supplementary Figure 1**). For standardized analysis by high-dimensional mass cytometry, 500 µL aliquots of whole blood samples of healthy controls and SLE patients were thawed in batches, leukocytes were isolated, barcoded and pooled, and stained with pre-prepared antibody cocktails to resolve major blood immune cell subsets, as well as levels of phosphorylated signal transduction proteins (**Figure 1**). The latter were selected to cover a broad range of transducers involved in cytokine and antigen receptor-, as well as calcium- and MAPK-dependent signaling (**Figure 1**; **Supplementary Table 1**), which we hypothesized to be altered in SLE cell-intrinsically or in response to the chronic inflammatory environment.

### 3.2 UACT cell signaling patterns are cell type- and state-specific, and altered in SLE

Major blood leukocyte subsets were distinguished according to the expression of cell type- and state-defining markers (**Supplementary Figures 4**, **5**), yielding a total of 32 subsets for UACT analysis.

Phosphorylation patterns varied between major leukocyte lineages and subsets (**Supplementary Figure 6**), consistent with differences in cytokine receptor expression and cell type- or state-specific signaling. Related cell types, like neutrophils and eosinophils or B and T cells, showed more similar UACT profiles compared to more distantly related cell types such as monocytes. Plasmablasts and plasma cells (PB/PC) and monocytes exhibited the highest average phosphorylation signals across multiple proteins, consistent with the role of the latter as environmental sensors. Differentiation states of naïve and memory B, CD4, and CD8 T cells were associated with distinct UACT patterns, as were classical and nonclassical monocytes, and subsets of NK (natural killer) cells and DCs (dendritic cells). These findings highlight the cell type- and state-specific nature of UACT, supporting its utility for patient immune profiling.

**Figure 2:**
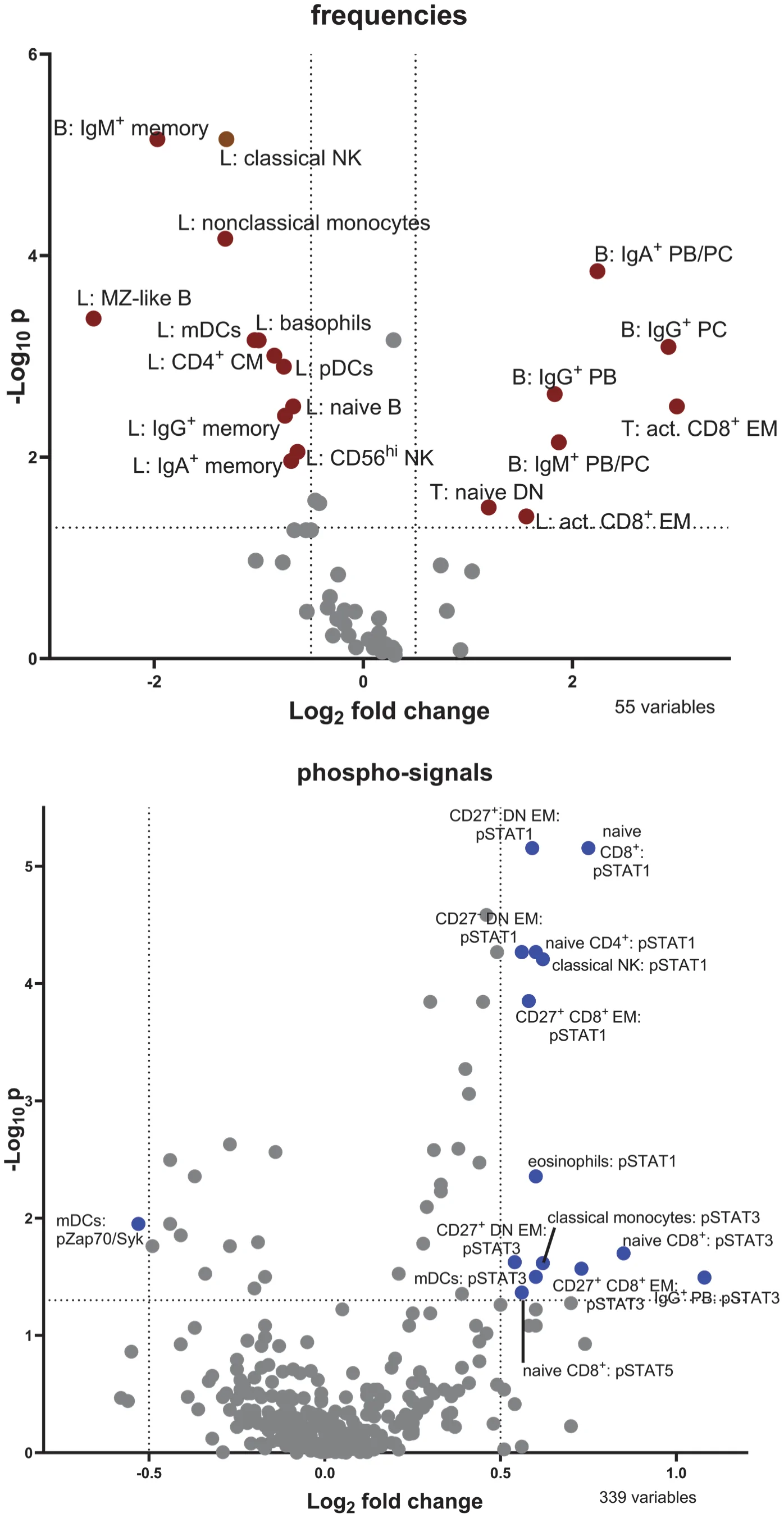
Cellular fingerprint of SLE. Volcano plots showing log2 fold changes of SLE vs. healthy controls plotted against -log10 p-values for cellular composition (top) and signal transducer phosphorylation (bottom). Frequencies are indicated as percent of CD45+ leukocytes (L), B cells (B) or T cells (T).

Building on this, we next analyzed disease-related changes in blood leukocyte composition and UACT in 20 patients with SLE, as well as their association with disease activity or clinical manifestations. 15 out of 20 patients (75%) presented with active disease as defined by a SLEDAI-2K score greater than or equal to 6 (mean 8.5) and diverse clinical manifestations and autoantibody profiles (**Table 1**; **Supplementary Tables 2**, **3**). Our cohort presented with known SLE-associated aberrations in blood leukocyte composition, such as selective leukopenia, altered B cell composition (56), decreased dendritic cells (57), nonclassical monocytes (58), or basophils (59), as well as an expansion of plasmablasts and plasma cells (PB/PC) (34, 60, 61) and of activated HLA-DR+ CD38+ T cells (62) (**Figure 2**).

When analyzing disease-related changes in UACT, increased pSTAT levels in naïve T cells were among the most significantly altered features in SLE (**Figure 2**). pSTAT1 levels were increased in naïve CD4 and CD8 T cells (1.5- and 1.7-fold) and pSTAT3 and 5 signals were found to be increased (1.8- and 1.5-fold) in naïve CD8 T cells compared to controls. Beyond that, SLE patients showed an increase in pSTAT1 levels across selected innate and adaptive immune cell populations, especially eosinophils, classical NK cells and CD8 and double-negative (DN) T cells (approx. 1.5-fold). Additionally, pSTAT3 was increased in classical monocytes and myeloid DCs (mDC) (both 1.5-fold), IgG+ PB (2.1-fold) and CD27+ CD8 and DN memory T cell subsets (1.7- and 1.5-fold). Notably, significant changes in SLE patients on the group level were limited to differences in pSTAT levels. However, single patients presented with other signaling changes, such as high levels of pp38 in B and plasmablast populations. This underlines the sensitivity of UACT to detect *in vivo*-induced changes of signal transducers beyond JAK-STAT signaling and supports the potential utility of our approach for patient stratification.

To identify potential inducers of the altered cell signaling states we observed, we performed a bead-based cytokine assay to quantify serum levels of 45 cytokines and chemokines in our SLE cohort. As pSTAT1,3, and 5 are inducible by type I IFN, as well as other disease-relevant cytokines such as IL-10, IL-12, or IL-23, we aimed to assess whether pSTAT levels were indeed positively correlated serum cytokine levels. Correlation of pSTAT levels with serum levels of IFNα, which is notoriously difficult to measure (32), could not be reliably assessed because they were close to or below the detection limit in our assay. Signals of other cytokines such as MCP-1 (CCL-2), IL-18, IL-5, IFNγ and IP-10 (CXCL-10), however, were found to correlate with pSTAT1 and/or 3 signals across T cell subsets and innate immune cells such as monocytes, mDCs, and NK cells (**Supplementary Figure 7**). While some of these cytokines, such as IFNγ or IL-5, can directly induce JAK-STAT signaling, some of the correlations are likely a secondary effect of the increased pro-inflammatory cytokine production in response to the original inflammatory stimulus. This is supported by the fact that the levels of these cytokines were positively correlated with each other, and MCP-1 and IP-10 expression can be induced by IFN. Because serum IFNα could not be reliably quantified in our assay, we cannot directly confirm type I IFN as the driver of the pSTAT signatures observed here, although we also rely on SIGLEC-1 as another type I activation surrogate marker. The patterns are consistent with, but not proof of, type I IFN pathway activation, and other cytokines capable of inducing STAT phosphorylation (e.g., IFNγ, IL-10, IL-12, IL-23) cannot be excluded as contributors. We also note that the statistical significance of the findings was established based on bootstrapping and that this analysis should be considered exploratory.

### 3.3 pSTAT1 and 3 levels in distinct T cell subsets are associated with immunological markers of SLE

Because we observed considerable patient variability regarding UACT, we subsequently explored correlations of these characteristics with clinical and immunological parameters.

**Figure 3:**
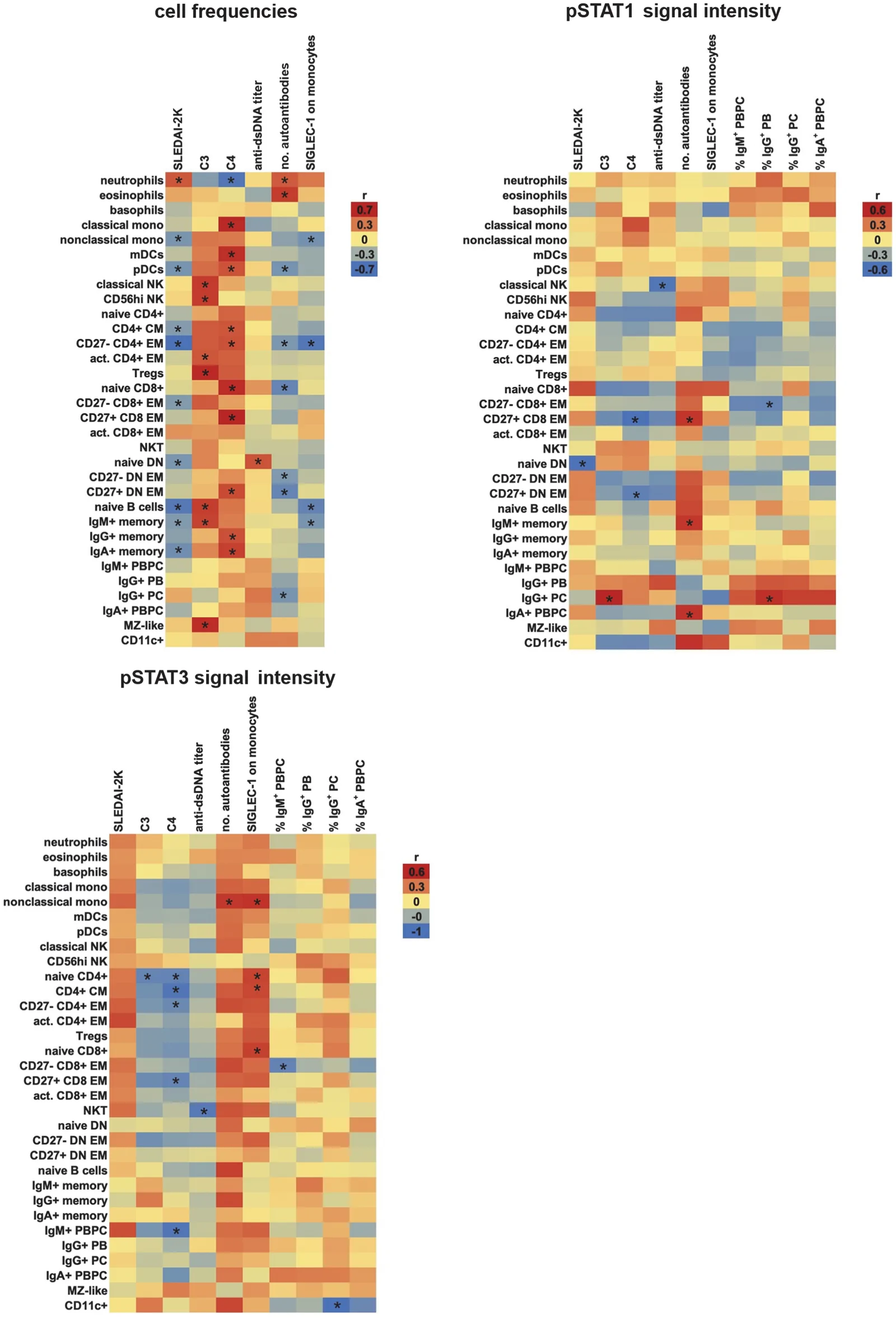
Correlation of cellular frequencies and phospho-proteins with clinical and immunological hallmarks of SLE. Cell frequencies (as percent of leukocytes) and pSTAT1 and 3 signals of individual subsets were correlated with clinical and immunological features of SLE. Spearman correlations coefficients (r) for 20 SLE patients are shown, and statistically significant correlations (non-adjusted p < 0.05) are marked with *.

In this exploratory analysis, pSTAT1 levels in CD27+ CD8 and DN effector memory T cell subsets were negatively correlated with complement C4 serum levels, and pSTAT1 in CD27+ CD8 memory T cells, IgM memory B cells and IgA PB/PC were positively correlated with the number of autoantibody specificities in patients (**Figure 3**). pSTAT3 levels in T cell subsets were also correlated with complement consumption, and pSTAT3 in naïve CD4 and CD8 T cells, CD4 central memory T cells and nonclassical monocytes was positively correlated with SIGLEC-1 expression on monocytes, which is associated with type I IFN activation.

When analyzing differences between clinical manifestations, we found that patients with proteinuria (n=5, **Supplementary Figure 8**) had higher pSTAT1 signals in naive CD8 T cells and CD27+ DN memory T cells, as well as higher pSTAT3 in basophils, NKT cells and IgM+ PB/PC. Patients with vasculitis (n=3, **Supplementary Figure 8**) showed increased pSTAT3 in basophils and IgM+ PB/PC compared to those without, although interpatient variability in this small group was relatively high. Patients with higher numbers of different autoantibody specificities generally showed higher levels of STAT phosphorylation than those who were positive for anti-dsDNA antibodies alone, but there was no clear trend with regards to their specificity (**Supplementary Figure 9**).

To exclude medication as a potential confounder, we compared patients taking prednisone, hydroxychloroquine, methotrexate, azathioprine or belimumab to those not receiving the respective treatment (**Supplementary Figure 10**). Patients taking prednisone had significantly higher pSTAT3 levels in mDCs, naive CD8 T cells and IgM+ PB/PC (**Supplementary Figure 10A**). Patients taking hydroxychloroquine (**Supplementary Figure 10B**) showed lower pSTAT3 levels in basophils, monocyte subsets, NKT cells and IgM+ PB/PC. However, patients undergoing prednisone therapy had higher disease activities (SLEDAI-2K) than those without (**Supplementary Table 2**), and patients not taking hydroxychloroquine had a higher incidence of severe SLE manifestations, such as nephritis, suggesting that disease activity, rather than the medication itself, may be the more likely driver of the observed differences. However, given the small sample size and overlap between treatment groups due to some patients taking multiple drug, we cannot formally exclude an independent medication effect.

### 3.4 UACT analysis improves separation of SLE vs. controls and identifies exploratory patient clusters with distinct clinical and immunological hallmarks of SLE

Next, we used multidimensional scaling (mds) on UACT data to evaluate its ability to separate diseased and healthy individuals compared to cell frequency data alone. Despite the complexity of individual protein- and donor phosphorylation signatures (**Supplementary Figures 11**, **12**), pSTAT provided the clearest group separation and best captured patient variability (**Supplementary Figure 13**), consistent with findings from comparisons at the group level (**Figure 2**). We therefore focused subsequent analyses on pSTAT UACT across 32 cell populations to assess its added value in differentiating SLE from controls and identifying patient subgroups.

**Figure 4:**
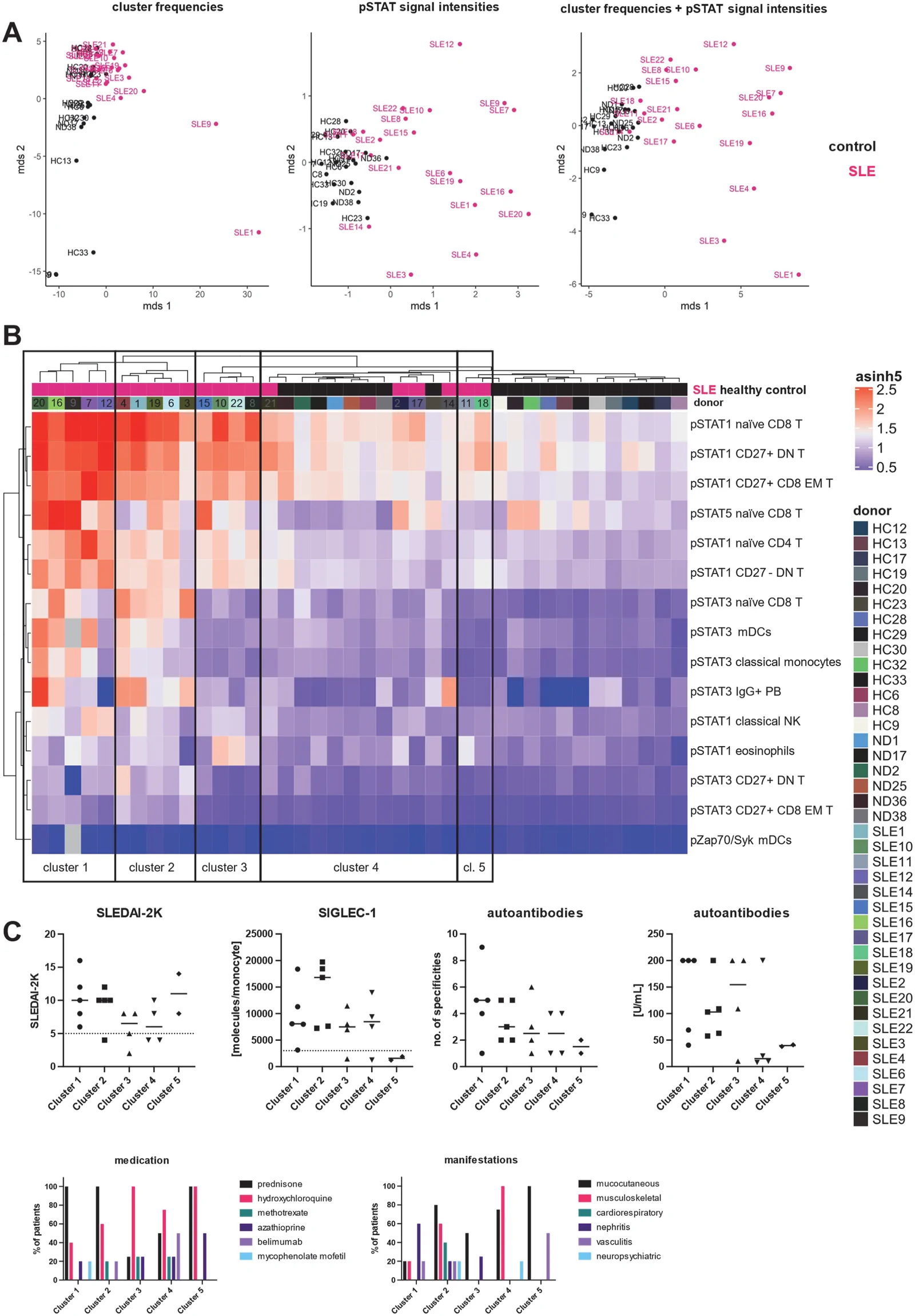
Dimension reduction and clustering of SLE patients based on cell signaling readouts. **(A)** Cluster frequency data (left), significantly different pSTAT1,3 and 5 mean signal intensity values (middle) and both (right) were used to calculate dimension reduction by multidimensional scaling (mds) for SLE patients (pink) and controls (black) to investigate the potential of each data type for classification and separation between the groups. To account for differences in the magnitude in numbers when dealing with cell frequency vs. signal intensity data, the values for the combined plots (right) were z-transformed prior to running the mds. **(B)** SLE patients and healthy controls were clustered according to their levels of the pSTAT1, 3 and 5 that were found to be significantly different between the two groups. Arcsinh-transformed mean intensity values are shown. Disease status and donor identity are indicated by numbers and color codes. **(C)** The five patient clusters identified in **(B)** were compared regarding their SLEDAI-2K disease activity scores, SIGLEC-1 expression on monocytes, number of autoantibody specificities, and titers of anti-doublestranded (ds)DNA autoantibodies. Additionally, the percentage of SLE patients in each cluster taking the indicated medication or suffering from the indicated clinical manifestations were analyzed (see also **Supplementary Tables 2**, **3**).

Cell frequency data alone could partially separate SLE patients from controls (**Figure 4**, left panel), especially along the mds1 axis, which seemed to be driven by lymphopenia, very low abundance of basophils, nonclassical monocytes, DCs and increased frequencies of HLA-DR+ CD38+ CD8 effector memory cells, as well as plasmablasts.

UACT readouts (**Figure 4**, middle and right) further improved separation between both groups along the mds1 and mds2 axes and pSTAT signal intensities seemed to better reflect SLE patient heterogeneity compared to cellular abundances. As expected, patients grouping furthest away from the healthy controls displayed very high pSTAT1, pSTAT3 and/or pSTAT5 signals across multiple immune cell subsets (**Figure 4**, middle). Clinically, they were characterized by high monocytic SIGLEC-1 expression, half of them had very high (>200 U/mL) anti-dsDNA autoantibody titers, and all except one were positive for at least one additional autoantibody specificity (e.g., Ro, La, Sm, RF, ACPA, U1RNP, RNP70, CENP) (**Supplementary Tables 2**, **3**).

Overall, UACT readouts added additional value in the distinction of SLE vs. controls and could thus be of interest for further investigation as diagnostic biomarkers.

To evaluate whether we could also discover distinct patient subgroups based on UACT, we next clustered our cohort according to their UACT patterns. For this analysis, we used only the most significantly altered signaling features of SLE (**Figure 2**), to reduce the input complexity and explore whether these UACT features could reveal patterns of patient heterogeneity. We note that this clustering is exploratory and intended to generate hypotheses about UACT-based patient stratification. Based on these 15 features, we identified 5 patient clusters with distinct combinatorics in their UACT in this exploratory cohort (**Figure 4**).

Patient cluster 1 was characterized by very high pSTAT1 and 3 levels across innate and T cell subsets, especially of pSTAT1 in T cell subsets, and comprised five patients, all of which had active disease and were positive for monocytic SIGLEC-1 expression. Three of the patients suffered from lupus nephritis, one from vasculitis, one from mucocutaneous and one from musculoskeletal symptoms. Four out of five patients were positive for four or more autoantibody specificities and three had very high (>200 U/mL) anti-dsDNA autoantibody titers (**Figure 4**). Taken together, patients with high UACT signals in cluster 1 tended to exhibit more active disease and a higher prevalence of severe manifestations.

Patient cluster 2 showed a similar, though less pronounced, UACT compared to cluster 1. This cluster also comprised five patients, but had a higher prevalence of mucocutaneous symptoms and musculoskeletal symptoms, as well as cardiovascular symptoms compared with cluster 1, but fewer patients with nephritis. All patients had high SIGLEC-1 expression on monocytes and all but one had active disease and moderate to high anti-dsDNA autoantibody titers.

Patient clusters 3,4,5 were more closely grouped with controls. Patient cluster 3, albeit closer to the controls in the mds and clustering analysis, still showed a distinct pSTAT1, but not pSTAT3 signature and contained four patients. Two had low disease activity (SLEDAI-2K < 6), three were SIGLEC-1- positive and three had moderate to high anti-dsDNA autoantibody titers. Two of the patients had mucocutaneous manifestations, and one had lupus nephritis. Cluster 4 contained four patients and was characterized by STAT1 phosphorylation in CD27+ memory T cells, as well as STAT1 and STAT5 UACT in naive CD8 T cells. Two had active disease (SLEDAI-2K > 6), three had high SIGLEC-1 on monocytes, three had mucocutaneous and four musculoskeletal manifestations. Finally, cluster 5 contained two patients with sporadic pSTAT1 UACT, but otherwise low phosphorylation levels compared to the other patients. Accordingly, these patients had also grouped with the controls in the mds plot. They had moderately high anti-dsDNA autoantibody titers, suffered from mucocutaneous symptoms and had active disease (SLEDAI-2K >6), but were SIGLEC-1-negative.

In summary, *in vivo*-like cell signaling analysis using UACT revealed heterogenous activation patterns among SLE patients and identified exploratory patient clusters associated with distinct clinical manifestations and immunological profiles within this cohort (**Supplementary Figure 14**). These findings point to the potential of UACT in resolving biologically relevant disease heterogeneity, but require further exploration and validation in larger independent cohorts.

### 3.5 Longitudinal UACT monitoring during baricitinib treatment points to a potential relationship between pSTAT3 baseline levels and clinical response

**Figure 5:**
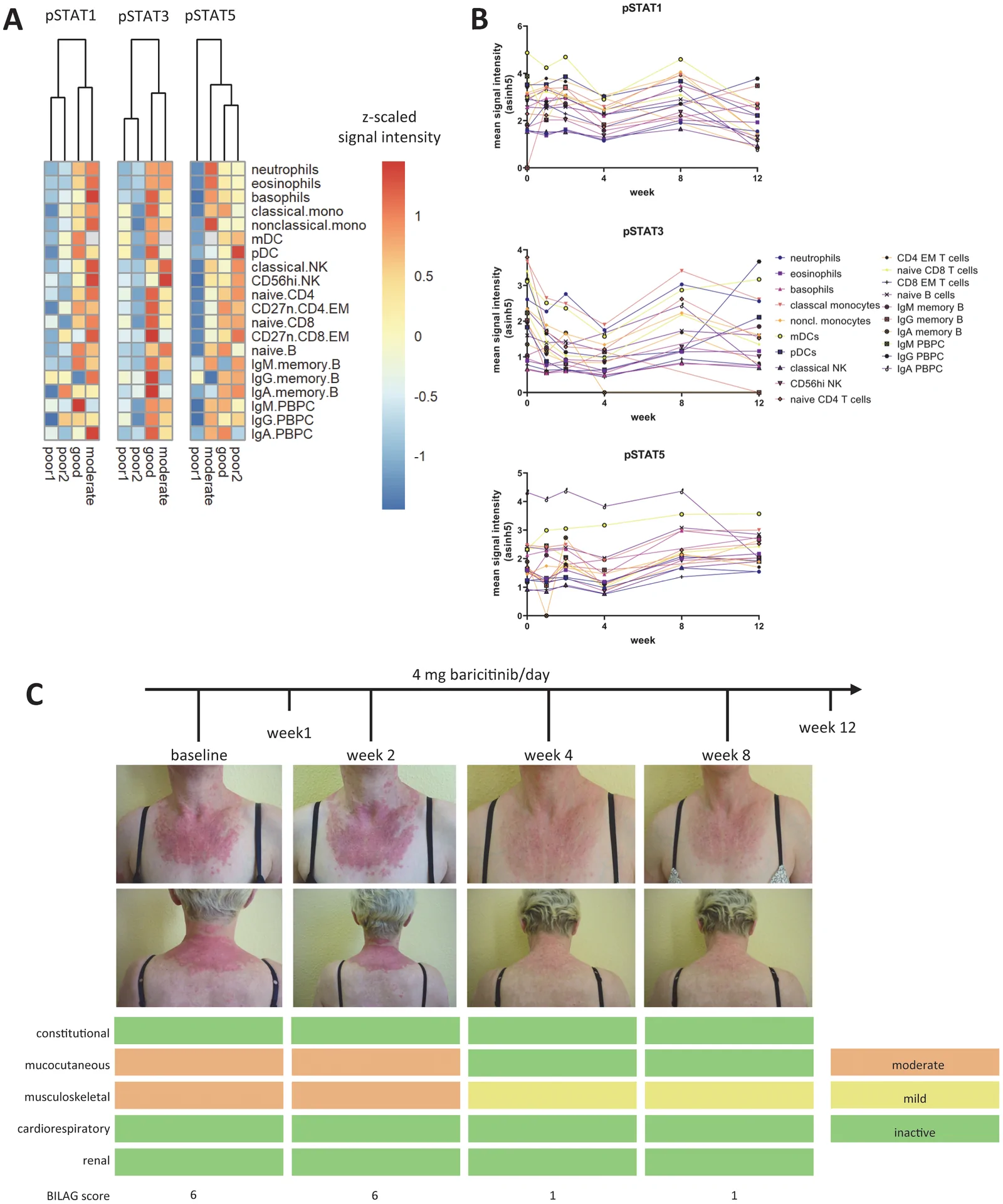
Association of pSTAT baseline levels with treatment responsiveness and pSTAT kinetics under treatment with baricitinib. **(A)** Four SLE patients treated with the JAK inhibitor baricitinib were grouped according to the quality of their treatment response and analyzed for baseline levels of pSTAT1,3,5 across different subsets. Subsets identified as part of the cellular fingerprint of SLE (**Figure 2**; or closely related ones) are shown here. **(B)** Kinetics of STAT1,3,5 phosphorylation across immune cell subsets of the good responder and **(C)** clinical responsiveness of the good responder under baricitinib treatment, as assessed by the physician based on improvement of clinical manifestations and BILAG-2004 scores. Images are shown with explicit patient consent.

Building on our findings that pSTAT UACT improves characterization of SLE patients, we explored its potential for treatment monitoring, another critical need in SLE management. In line with the importance of JAK-STAT signaling in SLE in response to many inflammatory cytokines, four patients with therapy-refractory skin manifestations received compassionate use treatment with baricitinib, a JAK1/2 inhibitor. UACT signatures of these patients were monitored at baseline and tracked during treatment to assess whether we could detect cellular treatment response and any association with clinical improvement. Based on the physician-assessed improvement of clinical manifestations and disease activity score BILAG-2004 (British Isles Lupus Assessment Group), one patient was classified as a good responder, one as a moderate responder, and two as non-responders. In this small exploratory cohort, the patient with the strongest clinical response had higher baseline pSTAT3 across almost all major immune cell subsets, as well as elevated pSTAT1 in DCs, B cells, and PB/PCs, while the moderate responder showed higher overall pSTAT1 levels, but less pronounced pSTAT3 activation, compared to the responder (**Figure 5A**).

Consistent with the successful signaling inhibition by baricitinib, pSTAT3 levels in the responder rapidly declined after treatment (**Figure 5B**, middle), paralleled by an improvement in clinical symptoms, especially the previously refractory skin manifestation (**Figure 5C**) and a reduction of the BILAG-2004 score from 6 at baseline to 1 at week 4 and after. pSTAT1 signals in this patient also decreased over time, but with much slower kinetics (**Figure 5B**, top), and overall, pSTAT1 and 5 were less affected by JAK inhibition than pSTAT3 across patients (**Supplementary Figures 15A–C**).

Taken together, UACT readouts were sufficiently sensitive to detect baricitinib-related decreases in STAT phosphorylation, making them an interesting tool for therapy monitoring in larger future studies.

## 4 Discussion

Our proof-of-concept study highlights that peripheral blood leukocytes can indeed act as living biosensors of the chronic inflammatory environment in SLE, and that the interrogation of their Unperturbed Activation Profiles (UACT) provides a window into the cells’ *in vivo* instruction by cell-intrinsic and extrinsic factors. We focused on capturing native *in vivo*-like pathway activity in patients, rather than stimulation-induced response capacity of cells to certain cytokines, to see if phosphorylation patterns could directly reveal inflammatory drivers and their imprints in individual patients which could be used to inform treatment choices.

This rationale is supported by previous cytometry studies on inflammatory diseases and cancer. In line with our findings, cytometric analysis of PBMCs from RA and SLE patients has shown that phosphorylation-based readouts can reveal important, cell-type specific information about signaling aberrations in chronic inflammatory conditions, and has also described increased pSTAT levels at baseline compared to healthy controls (37, 42, 63, 64). In line with this high intrinsic pre-activation, cells from patients with chronic inflammatory disease are often hyporesponsive compared to controls in stimulation experiments (38, 39). Similar observations of hyporesponsive STAT signaling in response to stimulation with IL-6 or IFN have also been made in cancer patients, and have been described to correlate with worse clinical outcomes (65, 66). In lymphoma, the presence of a subpopulation of B cells with impaired BCR signaling has been linked to worse survival, and signaling signatures in acute myeloid leukemia stratified patients into subgroups linked to chemotherapy treatment responsiveness (67, 68). Such results thus further support the utility of cell signaling-based, but not necessarily stimulation-based, biomarkers for improved patient stratification and treatment selection.

In this study, we applied our highly standardized UACT approach to SLE, a systemic autoimmune disease with broad immune system activation and considerable clinical heterogeneity, and thus a useful test case. In line with a strong role of type I IFN and other pro-inflammatory cytokines in SLE, we observed increased levels of pSTAT1 and 3 in multiple innate and adaptive immune cell populations, as well as an increase in pSTAT5 in naïve CD8 T cells. Overall, T cells displayed the most prominently altered UACT in patients, especially in the CD8 and DN compartment, and abnormalities in T cell signaling, metabolism, mitochondrial function and cytotoxic function of T cells have been described as an important feature in SLE pathology (69–72). UACT changes in most SLE patients largely affected pSTAT proteins, although individual donor signatures were complex. The pSTAT activation patterns and fold changes we detected with our UACT approach are consistent with the results of other cytometry-based studies in SLE and RA (37, 42, 63, 64), supporting the potential of UACT to detect meaningful disease-specific signaling alterations. A recent study using split samples and clone-matched antibody panels also showed that mass cytometry-based detection showed equal or better resolution of phospho-protein detection relative to (spectral) fluorescence-based cytometry, supporting mass cytometry as a valuable platform for UACT detection, at least at the discovery research stage that precedes potential translation into smaller, more readily applicable panels for clinical routine (73).

UACT readouts showed limited correlation with disease activity scores (SLEDAI-2K) or monocytic SIGLEC-1 expression. This may be partly due to the limitations of the SLEDAI-2K, which only reflects the presence, not the severity, of symptoms within the 30 days prior to the recording (74, 75). The weak association between phosphorylated signaling proteins and SIGLEC-1 may result from chronic cytokine stimulation leading to altered or dampened cellular responses (76). However, UACT was designed to identify individual inflammatory drivers, potential patient subtypes, and potential targeted treatment options, and not necessarily to reflect disease activity.

Notably, UACT readouts were able to separate SLE patients and healthy controls more clearly than cell abundance readouts in mds in this cohort. Furthermore, UACT signatures revealed heterogeneous signaling patterns among patients and enabled the identification of five exploratory patient clusters characterized by distinct combinations of pSTAT T cell UACT signatures, SIGLEC-1 expression as a surrogate marker for type I IFN activation, and clinical symptoms. Given the limited cohort size, particularly the small number of patients in some clusters, these findings should be considered hypothesis-generating and require validation in larger patient cohorts with different clinical outcomes. Nevertheless, similar observations have been reported in patients with lupus nephritis, where immune activation patterns in peripheral blood revealed three subgroups of patients with low immunological activity, and strong IFN or cytotoxic T cell signatures, respectively (77).

Overall, the observations in this proof-of-concept demonstrate the ability of our approach to capture aspects of the pro-inflammatory environment in patients and point to cell types and signaling pathways that could be of particular therapeutic relevance in SLE.

Given the central importance of JAK-STAT signaling in SLE, four patients with refractory skin manifestations were treated with the JAK1/2 inhibitor baricitinib under compassionate use. To assess whether our UACT system could also be used to monitor treatment success, we monitored these patients before and during treatment. The JAK inhibitor treatment led to a detectable reduction in pSTAT3 levels across multiple cell types within the first month. High levels of pSTAT3 at baseline seemed to be associated with favorable treatment response to JAK inhibition and the pattern of pSTAT3 decrease paralleled clinical improvement in one responder patient. In contrast, pSTAT1 and pSTAT5 seemed to be less consistently affected by baricitinib treatment, with variable responses across different cell types. These findings align with previous studies showing differential effects of JAK inhibitors on STAT signaling, depending on cell type and cytokine stimulus (78). Overall, results of clinical trials with baricitinib in SLE have been mixed so far, potentially due to patient heterogeneity in immune activation (79, 80). Although based on a very small number of patients, our observations indicate that pSTAT baseline levels, and UACT signatures more broadly, might of interest for future therapy monitoring studies and potential response prediction in larger cohorts of patients with inflammatory diseases.

Characterization of the lineage-, differentiation state- and disease-related UACT patterns was performed using functional high-dimensional mass cytometry. Our pipeline involves a quick, easy sample preservation step, as well as measures to standardize sample analysis and instrumentation, thus enabling highly standardized future studies of large cohorts involving thousands of patients and multiple recruitment sites. The fast, one-step fixation protocol has been optimized to allow for rapid, large-scale biobanking in clinical studies, without the need for sample pre-processing. Timely sample preservation is crucial to capture UACT, and high precision in sample collection and analysis is required, since *in vivo* phosphorylation signatures that can be subject to rapid degradation or perturbation by storage or processing. We deemed 30 minutes between blood collection and sample preservation to be an appropriate compromise between practical feasibility and appropriate signal preservation for the readouts included in this study. Workarounds like blood collection tubes pre-filled with appropriate fixatives could help to further facilitate and standardize timely sample preservation but will require further investigation.

The antibody panel used here was specifically designed to provide a broad overview over all compartments of blood immune cells (47), as it maximizes complex information from small sample volumes (500 µL of blood or less), e.g., from pediatric patients or rare sample specimens and could, through appropriate study setups and modeling even be used for the delineation of similarities, as well as potential interactions and links between signaling activation patterns of different cell populations. When specific cell subsets or signaling pathways of interest have been identified with our screening approach, follow up studies with specialized panels or larger sample volumes can easily be added for complementary analysis. Some fixation-sensitive epitopes like chemokine or cytokine receptors, however, might not be compatible with the current UACT protocol and their detection in the same assay would need to be further optimized.

Cell activation patterns observed in blood can only approximate the inflammatory processes taking place in the affected tissues, and drivers of tissue pathology may vary between organs. The UACT signatures of recirculating cells might therefore reflect averages or mixed signals derived from different tissues, although transcriptomic analyses have found good overall correlation between blood and tissue signatures (27). While markers of cellular homing and migratory capacity were not yet included in this first study, we have paved the way for future UACT studies to identify reliable biomarkers linked to certain organ manifestations or treatment responsiveness in systemic autoimmune diseases through side-by-side comparisons of blood and tissue. Nevertheless, peripheral blood as a transport medium for disease-instructed leukocytes remains of major importance for analyzing and monitoring cell-based biomarkers, and is most compatible with the demands of routine diagnostic purposes.

Taken together, our work has detected consistent cell signaling alterations that distinguished SLE patients from controls and revealed heterogenous UACT signatures associated with differences in disease manifestations and autoantibody profiles in this exploratory cohort, as well as capturing changes in response to signaling inhibition treatment. While these results should be treated as hypothesis-generating, they highlight UACT signatures as an interesting approach to read out the cells’ “logbook” of their exposure to certain inflammatory environments which could thus help to gain valuable insights on individual divers of pathology by using a patient’s cells as biosensors.

Overall, this proof-of-concept study reports preliminary, hypothesis-generating results, as it is limited by a small cohort size, potential confounding effects of medications, and lack of independent validation of the UACT patterns. Studies with larger, clinically well-characterized patient cohorts are thus now needed to assess whether these UACT patterns indeed reflect reproducible, long-term, distinct subtypes of patients with clear differences in disease manifestations and immune activation mechanisms, and thus distinct therapeutic needs. Similar validation is needed for a potential association of pSTAT3 levels at baseline and responsiveness to baricitinib. While such future work is still necessary to evaluate the robustness, clinical relevance, and utility of these signatures for patient classification, treatment monitoring, or biomarker development, the proof-of-concept presented here highlights that UACT patterns might be of interest for such clinical and translational research settings.

Ultimately, future larger-scale UACT screenings may enable us to identify more concise signatures consisting of a few robust phosphorylation readouts in larger, readily detectable immune cell populations that act as biosensors for the inflammatory environment in individual patients. Such readily available, low-parameter panels could then be more easily implemented in clinical routine diagnostics and finally be able to shift the paradigm from a symptom-based diagnosis of chronic inflammatory disease to one that is based on the molecular immune cell activation profile of each individual patient, bringing rheumatology one step closer to precision medicine.

## Data Availability

The original contributions presented in the study are included in the article/**Supplementary Material**. Further inquiries can be directed to the corresponding author. The data generated for this study can be found at flowrepository.org, data set ID: FR-FCM-Z5UD.

## References

[1] Van Der Heijde D Daikh DI Betteridge N Burmester GR Hassett AL Matteson EL . Common language description of the term rheumatic and musculoskeletal diseases (RMDs) for use in communication with the lay public, healthcare providers and other stakeholders endorsed by the European League Against Rheumatism (EULAR) and the American College of Rheumatology (ACR). Ann Rheum Dis. (2018) 77:829–32. doi: 10.1136/annrheumdis-2017-212565 29525777

[2] Petri M Genovese M Engle E Hochberg M . Definition, incidence, and clinical description of flare in systemic lupus erythematosus. A prospective cohort study. Arthritis Rheum. (1991) 34:937–44. doi: 10.1002/art.1780340802 1859487

[3] Bykerk VP Shadick N Frits M Bingham CO Jeffery I Iannaccone C . Flares in rheumatoid arthritis: Frequency and management. A report from the BRASS Registry. J Rheumatol. (2014) 41:227–34. doi: 10.3899/jrheum.121521 24334643

[4] Sparks JA Costenbader KH . Genetics, environment, and gene-environment interactions in the development of systemic rheumatic diseases. Rheum Dis Clin N Am. (2014) 40:637–57. doi: 10.1016/j.rdc.2014.07.005 25437282 PMC4250576

[5] Teruel M Alarcón-Riquelme ME . The genetic basis of systemic lupus erythematosus: What are the risk factors and what have we learned. J Autoimmun. (2016) 74:161–75. doi: 10.1016/j.jaut.2016.08.001 27522116

[6] Graham DSC Akil M Vyse TJ . Association of polymorphisms across the tyrosine kinase gene, TYK2 in UK SLE families. Rheumatology. (2007) 46:927–30. doi: 10.1093/rheumatology/kel449 17384181

[7] Hussein HM Rahal EA . The role of viral infections in the development of autoimmune diseases. Crit Rev Microbiol. (2019) 45:394–412. doi: 10.1080/1040841X.2019.1614904 31145640

[8] Edwards JCW Szczepański L Szechiński J Filipowicz-Sosnowska A Emery P Close DR . Efficacy of B-cell–targeted therapy with rituximab in patients with rheumatoid arthritis. N Engl J Med. (2004) 350:2572–81. doi: 10.1056/NEJMoa032534 15201414

[9] Looney RJ Anolik JH Campbell D Felgar RE Young F Arend LJ . B cell depletion as a novel treatment for systemic lupus erythematosus: A phase I/II dose‐escalation trial of rituximab. Arthritis Rheum. (2004) 50:2580–9. doi: 10.1002/art.20430 15334472

[10] Hiepe F Radbruch A . Plasma cells as an innovative target in autoimmune disease with renal manifestations. Nat Rev Nephrol. (2016) 12:232–40. doi: 10.1038/nrneph.2016.20 26923204

[11] Ostendorf L Burns M Durek P Heinz GA Heinrich F Garantziotis P . Targeting CD38 with daratumumab in refractory systemic lupus erythematosus. N Engl J Med. (2020) 383:1149–55. doi: 10.1056/NEJMoa2023325 32937047

[12] Burns M Ostendorf L Biesen R Grützkau A Hiepe F Mei HE . Dysregulated CD38 expression on peripheral blood immune cell subsets in SLE. Int J Mol Sci. (2021) 22:2424. doi: 10.3390/ijms22052424 33670902 PMC7957821

[13] Keystone EC Kavanaugh AF Sharp JT Tannenbaum H Hua Y Teoh LS . Radiographic, clinical, and functional outcomes of treatment with adalimumab (a human anti–tumor necrosis factor monoclonal antibody) in patients with active rheumatoid arthritis receiving concomitant methotrexate therapy: A randomized, placebo‐controlled, 52‐week trial. Arthritis Rheum. (2004) 50:1400–11. doi: 10.1002/art.20217 15146409

[14] Smolen JS Beaulieu A Rubbert-Roth A Ramos-Remus C Rovensky J Alecock E . Effect of interleukin-6 receptor inhibition with tocilizumab in patients with rheumatoid arthritis (OPTION study): a double-blind, placebo-controlled, randomised trial. Lancet. (2008) 371:987–97. doi: 10.1016/S0140-6736(08)60453-5 18358926

[15] Wallace D Navarra S Petri M Gallacher A Thomas M Furie R . Safety profile of belimumab: pooled data from placebo-controlled phase 2 and 3 studies in patients with systemic lupus erythematosus. Lupus. (2013) 22:144–54. doi: 10.1177/0961203312469259 23213069

[16] Vital EM Merrill JT Morand EF Furie RA Bruce IN Tanaka Y . Anifrolumab efficacy and safety by type I interferon gene signature and clinical subgroups in patients with SLE: post hoc analysis of pooled data from two phase III trials. Ann Rheum Dis. (2022) 81:951–61. doi: 10.1136/annrheumdis-2021-221425 35338035 PMC9213795

[17] Aringer M Costenbader K Daikh D Brinks R Mosca M Ramsey‐Goldman R . 2019 European League Against Rheumatism/American College of Rheumatology classification criteria for systemic lupus erythematosus. Arthritis Rheumatol. (2019) 71:1400–12. doi: 10.1002/art.40930 31385462 PMC6827566

[18] Aletaha D Neogi T Silman AJ Funovits J Felson DT Bingham CO . 2010 Rheumatoid arthritis classification criteria: an American College of Rheumatology/European League Against Rheumatism collaborative initiative. Ann Rheum Dis. (2010) 69:1580–8. doi: 10.1136/ard.2010.138461 20699241

[19] Hyrich KL Watson KD Silman AJ Symmons DPM The BSR Biologics Register . Predictors of response to anti-TNF- therapy among patients with rheumatoid arthritis: results from the British Society for Rheumatology Biologics Register. Rheumatology. (2006) 45:1558–65. doi: 10.1093/rheumatology/kel149 16705046

[20] Silva-Fernandez L Hyrich K . When TNF inhibitors fail in RA—weighing up the options. Nat Rev Rheumatol. (2014) 10:262–4. doi: 10.1038/nrrheum.2014.34 24614594

[21] Kim M Merrill J Kalunian K Hahn B Roach A Izmirly P . Brief report: Longitudinal patterns of response to standard of care therapy for systemic lupus erythematosus: Implications for clinical trial design. Arthritis Rheumatol. (2017) 69:785–90. doi: 10.1002/art.40013 27992696

[22] Daha NA Toes REM . Are ACPA-positive and ACPA-negative RA the same disease? Nat Rev Rheumatol. (2011) 7:202–3. doi: 10.1038/nrrheum.2011.28 21455249

[23] Liu Z Davidson A . Taming lupus—a new understanding of pathogenesis is leading to clinical advances. Nat Med. (2012) 18:871–82. doi: 10.1038/nm.2752 22674006 PMC3607103

[24] Banchereau R Hong S Cantarel B Baldwin N Baisch J Edens M . Personalized immunomonitoring uncovers molecular networks that stratify lupus patients. Cell. (2016) 165:551–65. doi: 10.1016/j.cell.2016.03.008 27040498 PMC5426482

[25] Toro-Domínguez D Carmona-Sáez P Alarcón-Riquelme ME . Shared signatures between rheumatoid arthritis, systemic lupus erythematosus and Sjögren’s syndrome uncovered through gene expression meta-analysis. Arthritis Res Ther. (2014) 16:489. doi: 10.1186/s13075-014-0489-x 25466291 PMC4295333

[26] Toro-Domínguez D Lopez-Domínguez R García Moreno A Villatoro-García JA Martorell-Marugán J Goldman D . Differential treatments based on drug-induced gene expression signatures and longitudinal systemic lupus erythematosus stratification. Sci Rep. (2019) 9:15502. doi: 10.1038/s41598-019-51616-9 31664045 PMC6820741

[27] Higgs BW Liu Z White B Zhu W White WI Morehouse C . Patients with systemic lupus erythematosus, myositis, rheumatoid arthritis and scleroderma share activation of a common type I interferon pathway. Ann Rheum Dis. (2011) 70:2029–36. doi: 10.1136/ard.2011.150326 21803750

[28] Barturen G Babaei S Català‐Moll F Martínez‐Bueno M Makowska Z Martorell‐Marugán J . Integrative analysis reveals a molecular stratification of systemic autoimmune diseases. Arthritis Rheumatol. (2021) 73:1073–85. doi: 10.1002/art.41610 33497037

[29] Reynolds JA McCarthy EM Haque S Ngamjanyaporn P Sergeant JC Lee E . Cytokine profiling in active and quiescent SLE reveals distinct patient subpopulations. Arthritis Res Ther. (2018) 20:173. doi: 10.1186/s13075-018-1666-0 30092845 PMC6085716

[30] Pacheco Y Barahona-Correa J Monsalve DM Acosta-Ampudia Y Rojas M Rodríguez Y . Cytokine and autoantibody clusters interaction in systemic lupus erythematosus. J Transl Med. (2017) 15:239. doi: 10.1186/s12967-017-1345-y 29178890 PMC5702157

[31] De Jager W Bourcier K Rijkers GT Prakken BJ Seyfert-Margolis V . Prerequisites for cytokine measurements in clinical trials with multiplex immunoassays. BMC Immunol. (2009) 10:52. doi: 10.1186/1471-2172-10-52 19785746 PMC2761376

[32] Biesen R Demir C Barkhudarova F Grün JR Steinbrich‐Zöllner M Backhaus M . Sialic acid–binding Ig‐like lectin 1 expression in inflammatory and resident monocytes is a potential biomarker for monitoring disease activity and success of therapy in systemic lupus erythematosus. Arthritis Rheum. (2008) 58:1136–45. doi: 10.1002/art.23404 18383365

[33] Rose T Grützkau A Hirseland H Huscher D Dähnrich C Dzionek A . IFNα and its response proteins, IP-10 and SIGLEC-1, are biomarkers of disease activity in systemic lupus erythematosus. Ann Rheum Dis. (2013) 72:1639–45. doi: 10.1136/annrheumdis-2012-201586 23117242

[34] Jacobi AM Mei H Hoyer BF Mumtaz IM Thiele K Radbruch A . HLA-DRhigh/CD27high plasmablasts indicate active disease in patients with systemic lupus erythematosus. Ann Rheum Dis. (2010) 69:305–8. doi: 10.1136/ard.2008.096495 19196727

[35] O’Gorman WE Hsieh EWY Savig ES Gherardini PF Hernandez JD Hansmann L . Single-cell systems-level analysis of human Toll-like receptor activation defines a chemokine signature in patients with systemic lupus erythematosus. J Allergy Clin Immunol. (2015) 136:1326–36. doi: 10.1016/j.jaci.2015.04.008 26037552 PMC4640970

[36] O’Gorman WE Kong DS Balboni IM Rudra P Bolen CR Ghosh D . Mass cytometry identifies a distinct monocyte cytokine signature shared by clinically heterogeneous pediatric SLE patients. J Autoimmun. (2017) 81:74–89. doi: 10.1016/j.jaut.2017.03.010 28389038 PMC5628110

[37] Huang X Guo Y Bao C Shen N . Multidimensional single cell based STAT phosphorylation profiling identifies a novel biosignature for evaluation of systemic lupus erythematosus activity. PloS One. (2011) 6:e21671. doi: 10.1371/journal.pone.0021671 21799742 PMC3142107

[38] Weißenberg SY Szelinski F Schrezenmeier E Stefanski AL Wiedemann A Rincon-Arevalo H . Identification and characterization of post-activated B cells in systemic autoimmune diseases. Front Immunol. (2019) 10:2136. doi: 10.3389/fimmu.2019.02136 31616406 PMC6768969

[39] Slight-Webb S Smith M Bylinska A Macwana S Guthridge C Lu R . Autoantibody-positive healthy individuals with lower lupus risk display a unique immune endotype. J Allergy Clin Immunol. (2020) 146:1419–33. doi: 10.1016/j.jaci.2020.04.047 32446964 PMC7680268

[40] Rubin SJS Bai L Haileselassie Y Garay G Yun C Becker L . Mass cytometry reveals systemic and local immune signatures that distinguish inflammatory bowel diseases. Nat Commun. (2019) 10:2686. doi: 10.1038/s41467-019-10387-7 31217423 PMC6584653

[41] Sigal N Maecker HT . Mass cytometry assessment of cell phenotypes and signaling states in human whole blood, in: Apoptosis and Cancer (2022). New York, NY: Springer US. Available online at: https://link.springer.com/10.1007/978-1-0716-2553-8_10 (Accessed December 14, 2025). 10.1007/978-1-0716-2553-8_10 PMC9991871 36087263

[42] Macaubas C Bayram B Hussein N Jager A Davis KL Graf J . Using mass cytometry to probe the STAT signaling landscape in circulating immune cells in rheumatoid arthritis uncovers signaling dysregulation and correlation with disease activity. Front Med. (2025) 12:1622537. doi: 10.3389/fmed.2025.1622537 41446843 PMC12722927

[43] Wahren-Herlenius M Dörner T . Immunopathogenic mechanisms of systemic autoimmune disease. Lancet. (2013) 382:819–31. doi: 10.1016/S0140-6736(13)60954-X 23993191

[44] Tsokos GC . Systemic lupus erythematosus. N Engl J Med. (2011) 365:2110–21. doi: 10.1056/NEJMra1100359 22129255

[45] Zunder ER Finck R Behbehani GK Amir ED Krishnaswamy S Gonzalez VD . Palladium-based mass tag cell barcoding with a doublet-filtering scheme and single-cell deconvolution algorithm. Nat Protoc. (2015) 10:316–33. doi: 10.1038/nprot.2015.020 25612231 PMC4347881

[46] Rahman AH Tordesillas L Berin MC . Heparin reduces nonspecific eosinophil staining artifacts in mass cytometry experiments. Cytometry A. (2016) 89:601–7. doi: 10.1002/cyto.a.22826 27061608 PMC4919163

[47] Baumgart S Peddinghaus A Schulte‐Wrede U Mei HE Grützkau A . OMIP‐034: Comprehensive immune phenotyping of human peripheral leukocytes by mass cytometry for monitoring immunomodulatory therapies. Cytometry A. (2017) 91:34–8. doi: 10.1002/cyto.a.22894 27362704

[48] Schulz AR Baumgart S Schulze J Urbicht M Grützkau A Mei HE . Stabilizing antibody cocktails for mass cytometry. Cytometry A. (2019) 95:910–6. doi: 10.1002/cyto.a.23781 31058420

[49] Schulz AR Mei HE . Surface barcoding of live PBMC for multiplexed mass cytometry, in: Mass Cytometry (2019). New York, NY: Springer New York. Available online at: http://link.springer.com/10.1007/978-1-4939-9454-0_7 (Accessed September 14, 2025). 10.1007/978-1-4939-9454-0_7 31077101

[50] Chevrier S Crowell HL Zanotelli VRT Engler S Robinson MD Bodenmiller B . Compensation of signal spillover in suspension and imaging mass cytometry. Cell Syst. (2018) 6:612–620.e5. doi: 10.1016/j.cels.2018.02.010 29605184 PMC5981006

[51] Budzinski L Schulz AR Baumgart S Burns T Rose T Hirseland H . Osmium-labeled microspheres for bead-based assays in mass cytometry. J Immunol. (2019) 202:3103–12. doi: 10.4049/jimmunol.1801640 30988119

[52] Schuyler RP Jackson C Garcia-Perez JE Baxter RM Ogolla S Rochford R . Minimizing batch effects in mass cytometry data. Front Immunol. (2019) 10:2367. doi: 10.3389/fimmu.2019.02367 31681275 PMC6803429

[53] Linderman GC Rachh M Hoskins JG Steinerberger S Kluger Y . Fast interpolation-based t-SNE for improved visualization of single-cell RNA-seq data. Nat Methods. (2019) 16:243–5. doi: 10.1038/s41592-018-0308-4 30742040 PMC6402590

[54] Weber LM Nowicka M Soneson C Robinson MD . diffcyt: Differential discovery in high-dimensional cytometry via high-resolution clustering. Commun Biol. (2019) 2:183. doi: 10.1038/s42003-019-0415-5 31098416 PMC6517415

[55] Nowicka M Krieg C Crowell HL Weber LM Hartmann FJ Guglietta S . CyTOF workflow: differential discovery in high-throughput high-dimensional cytometry datasets. F1000Research. (2019) 6:748. doi: 10.12688/f1000research.11622.3 28663787 PMC5473464

[56] Odendahl M Jacobi A Hansen A Feist E Hiepe F Burmester GR . Disturbed peripheral B lymphocyte homeostasis in systemic lupus erythematosus. J Immunol. (2000) 165:5970–9. doi: 10.4049/jimmunol.165.10.5970 11067960

[57] Migita K Miyashita T Maeda Y Kimura H Nakamura M Yatsuhashi H . Reduced blood BDCA-2+ (lymphoid) and CD11c+ (myeloid) dendritic cells in systemic lupus erythematosus. Clin Exp Immunol. (2005) 142:84–91. doi: 10.1111/j.1365-2249.2005.02897.x 16178860 PMC1809479

[58] Ishii A Nakayamada S Ohkubo N Miyazaki Y Iwata S Annan J . Reduction of non-classical monocytes that suppress interferon-α in patients with systemic lupus erythematosus. Clin Exp Rheumatol. (2025) 43(9):1568–76. doi: 10.55563/clinexprheumatol/ziktdx 40314995

[59] Pan Q Gong L Xiao H Feng Y Li L Deng Z . Basophil activation-dependent autoantibody and interleukin-17 production exacerbate systemic lupus erythematosus. Front Immunol. (2017) 8. doi: 10.3389/fimmu.2017.00348 28396669 PMC5366357

[60] Jacobi AM Odendahl M Reiter K Bruns A Burmester GR Radbruch A . Correlation between circulating CD27^high^ plasma cells and disease activity in patients with systemic lupus erythematosus. Arthritis Rheum. (2003) 48:1332–42. doi: 10.1002/art.10949 12746906

[61] Speidel K Cheng Q Khodadadi L Sinzinger B Martin J Beenken AE . Increased type I interferon activity with concurrent plasmablast expansion identifies systemic lupus erythematosus patients with poor outcomes. Int J Mol Sci. (2026) 27:2852. doi: 10.3390/ijms27062852 41898711 PMC13026582

[62] Fujita Y Nakayamada S Kubo S Miyazaki Y Sonomoto K Tanaka H . Association of peripheral CD8^+^ T cell activation with disease activity and treatment resistance in systemic lupus erythematosus. RMD Open. (2025) 11:e005122. doi: 10.1136/rmdopen-2024-005122 40010940 PMC11865784

[63] Yiu G Rasmussen TK Tsai BL Diep VK Haddon DJ Tsoi J . High interferon signature leads to increased STAT1/3/5 phosphorylation in PBMCs from SLE patients by single cell mass cytometry. Front Immunol. (2022) 13:833636. doi: 10.3389/fimmu.2022.833636 35185925 PMC8851522

[64] Galligan CL Siebert JC Siminovitch KA Keystone EC Bykerk V Perez OD . Multiparameter phospho-flow analysis of lymphocytes in early rheumatoid arthritis: implications for diagnosis and monitoring drug therapy. PloS One. (2009) 4:e6703. doi: 10.1371/journal.pone.0006703 19693272 PMC2724743

[65] Critchley-Thorne RJ Simons DL Yan N Miyahira AK Dirbas FM Johnson DL . Impaired interferon signaling is a common immune defect in human cancer. Proc Natl Acad Sci USA. (2009) 106:9010–5. doi: 10.1073/pnas.0901329106 19451644 PMC2690021

[66] Wang L Miyahira AK Simons DL Lu X Chang AY Wang C . IL6 signaling in peripheral blood T cells predicts clinical outcome in breast cancer. Cancer Res. (2017) 77:1119–26. doi: 10.1158/0008-5472.CAN-16-1373 27879265 PMC5334262

[67] Irish JM Hovland R Krutzik PO Perez OD Bruserud Ø Gjertsen BT . Single cell profiling of potentiated phospho-protein networks in cancer cells. Cell. (2004) 118:217–28. doi: 10.1016/j.cell.2004.06.028 15260991

[68] Irish JM Myklebust JH Alizadeh AA Houot R Sharman JP Czerwinski DK . B-cell signaling networks reveal a negative prognostic human lymphoma cell subset that emerges during tumor progression. Proc Natl Acad Sci. (2010) 107:12747–54. doi: 10.1073/pnas.1002057107 20543139 PMC2919949

[69] Katsuyama T Tsokos GC Moulton VR . Aberrant T cell signaling and subsets in systemic lupus erythematosus. Front Immunol. (2018) 9:1088. doi: 10.3389/fimmu.2018.01088 29868033 PMC5967272

[70] Buang N Tapeng L Gray V Sardini A Whilding C Lightstone L . Type I interferons affect the metabolic fitness of CD8+ T cells from patients with systemic lupus erythematosus. Nat Commun. (2021) 12:1980. doi: 10.1038/s41467-021-22312-y 33790300 PMC8012390

[71] Sharabi A Tsokos GC . T cell metabolism: new insights in systemic lupus erythematosus pathogenesis and therapy. Nat Rev Rheumatol. (2020) 16:100–12. doi: 10.1038/s41584-019-0356-x 31949287

[72] Tilstra JS Avery L Menk AV Gordon RA Smita S Kane LP . Kidney-infiltrating T cells in murine lupus nephritis are metabolically and functionally exhausted. J Clin Invest. (2018) 128:4884–97. doi: 10.1172/JCI120859 30130253 PMC6205402

[73] Cohen MJ Smith‐Mahoney EL Bailey M Wang L Tracey LJ Polanco L . Superior intracellular detection of cytokines, transcription factors, and phosphoproteins by CyTOF compared with fluorescent cytometry. Cytometry A. (2026) 109:167–79. doi: 10.1002/cytoa.70011 41803010

[74] Gladman DD Ibañez D Urowitz MB . Systemic lupus erythematosus disease activity index 2000. J Rheumatol. (2002) 29:288–91. doi: 10.3899/jrheum.130030 11838846

[75] Mikdashi J Nived O . Measuring disease activity in adults with systemic lupus erythematosus: the challenges of administrative burden and responsiveness to patient concerns in clinical research. Arthritis Res Ther. (2015) 17:183. doi: 10.1186/s13075-015-0702-6 26189728 PMC4507322

[76] Niesner U Albrecht I Janke M Doebis C Loddenkemper C Lexberg MH . Autoregulation of Th1-mediated inflammation by twist1. J Exp Med. (2008) 205:1889–901. doi: 10.1084/jem.20072468 18663125 PMC2525589

[77] Horisberger A Griffith A Keegan J Arazi A Pulford J Murzin E . Blood immunophenotyping identifies distinct kidney histopathology and outcomes in patients with lupus nephritis. J Clin Invest. (2025) 135:e181034. doi: 10.1172/JCI181034 40536813 PMC12352894

[78] Traves PG Murray B Campigotto F Galien R Meng A Di Paolo JA . JAK selectivity and the implications for clinical inhibition of pharmacodynamic cytokine signalling by filgotinib, upadacitinib, tofacitinib and baricitinib. Ann Rheum Dis. (2021) 80:865–75. doi: 10.1136/annrheumdis-2020-219012 33741556 PMC8237188

[79] Morand EF Vital EM Petri M Van Vollenhoven R Wallace DJ Mosca M . Baricitinib for systemic lupus erythematosus: a double-blind, randomised, placebo-controlled, phase 3 trial (SLE-BRAVE-I). Lancet. (2023) 401:1001–10. doi: 10.1016/S0140-6736(22)02607-1 36848918

[80] Petri M Bruce IN Dörner T Tanaka Y Morand EF Kalunian KC . Baricitinib for systemic lupus erythematosus: a double-blind, randomised, placebo-controlled, phase 3 trial (SLE-BRAVE-II). Lancet. (2023) 401:1011–19. doi: 10.1016/S0140-6736(22)02546-6 36848919

