# Supplementary material for: Single-cell Unperturbed Activation Profiling by mass cytometry captures in vivo immune signaling in SLE
Source: Front Immunol. 2026 Aug 17;17:1844288. doi: 10.3389/fimmu.2026.1844288 (PMC13526989; doi:10.3389/fimmu.2026.1844288)
Supplement: Supplementary file 1 [file DataSheet1.pdf]

Supplementary Material

1 Supplementary Figures and Tables

1.1 Supplementary Figures

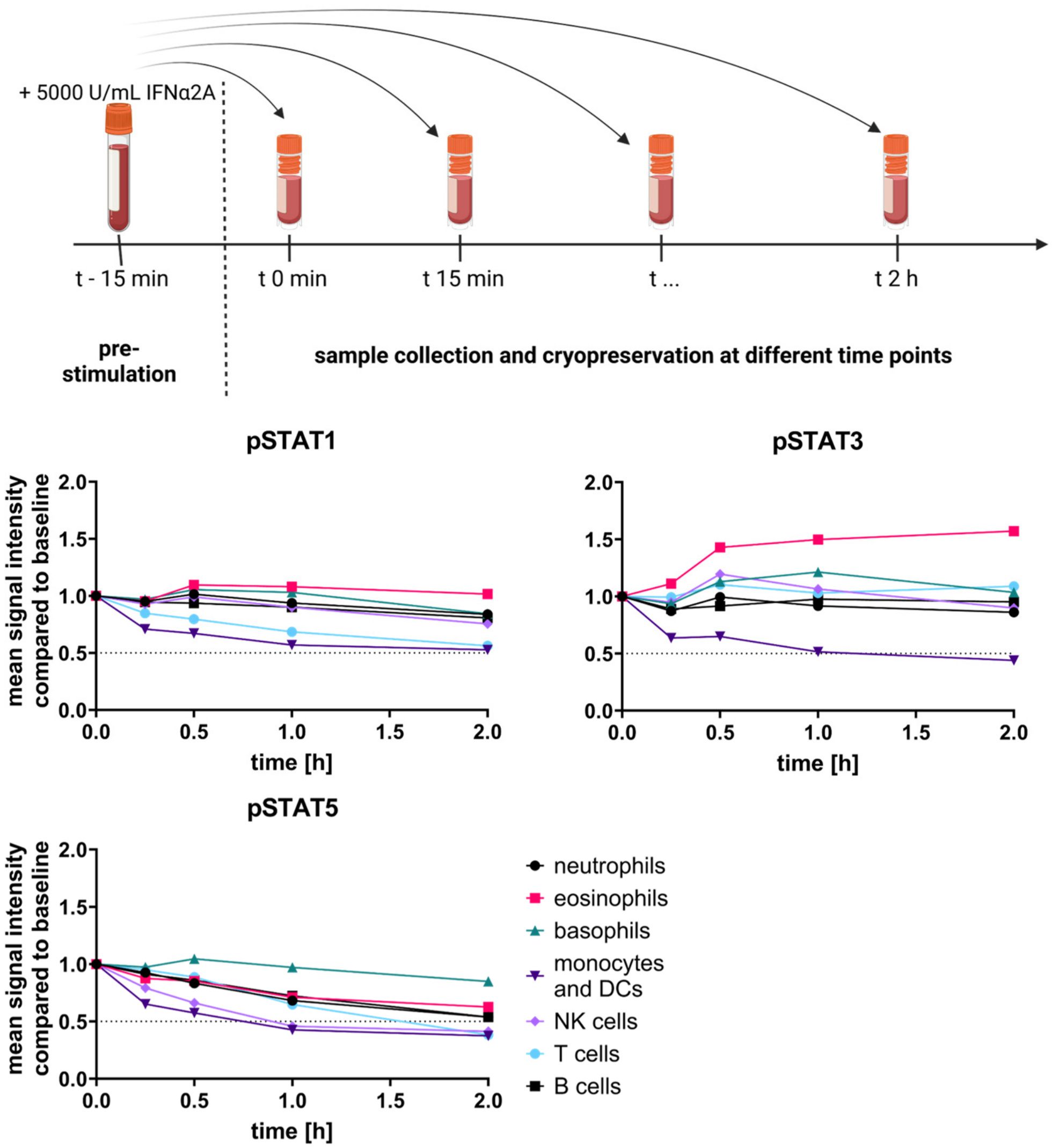

Figure S1: Kinetics of phospho-protein detectability in whole blood leukocyte subsets. To determine how long after blood collection in vivo induced protein phosphorylation could reliably be detected, we stimulated whole blood from a control donor with 5000 U / mL mL IFNα2A for 15 minutes, and then collected and cryopreserved aliquots from the bulk sample at the indicated time points. Average levels of pSTAT1, 3 and 5 signals were determined in various leukocyte subsets and

normalized to baseline values (t = 0 minutes). Schema at the top created in BioRender. Burns, M. (2026) https://BioRender.com/26zylyy

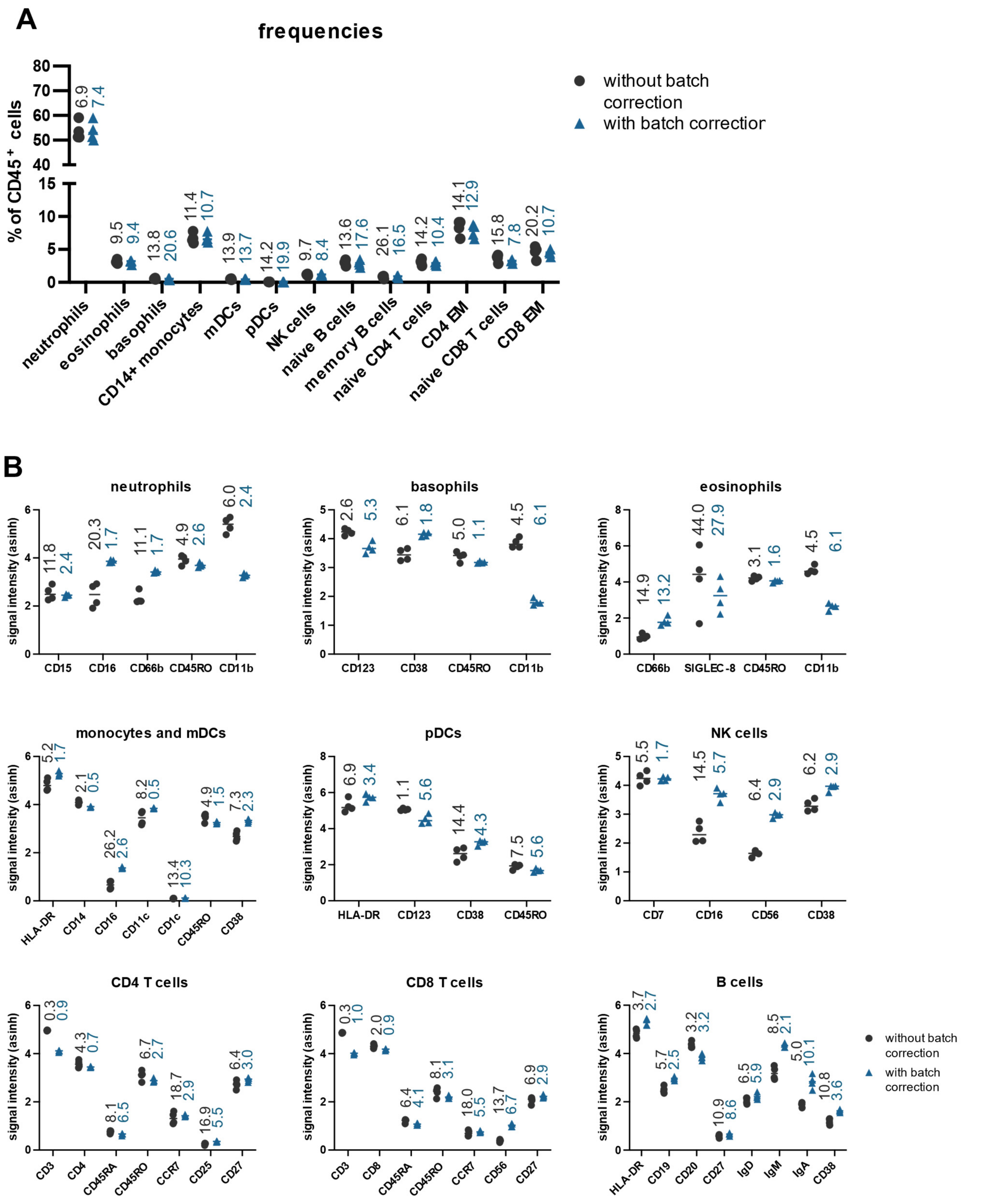

Figure S2: Effects of batch effect correction on cell abundance, surface marker and phospho-protein levels of reference samples (part 1/2). Frequencies **(A)**, lineage marker expression **(B)**, and pSTAT1, 3 and 5 levels **(C)** of major immune cell subsets in the references samples included in each study day were assessed before and after batch correction. Numbers represent coefficients of

variation (CV). One dot represents one reference sample. While absolute intensity values changed, no significant impact of batch correction on group differences between SLE and healthy controls was found (see Figure S3).

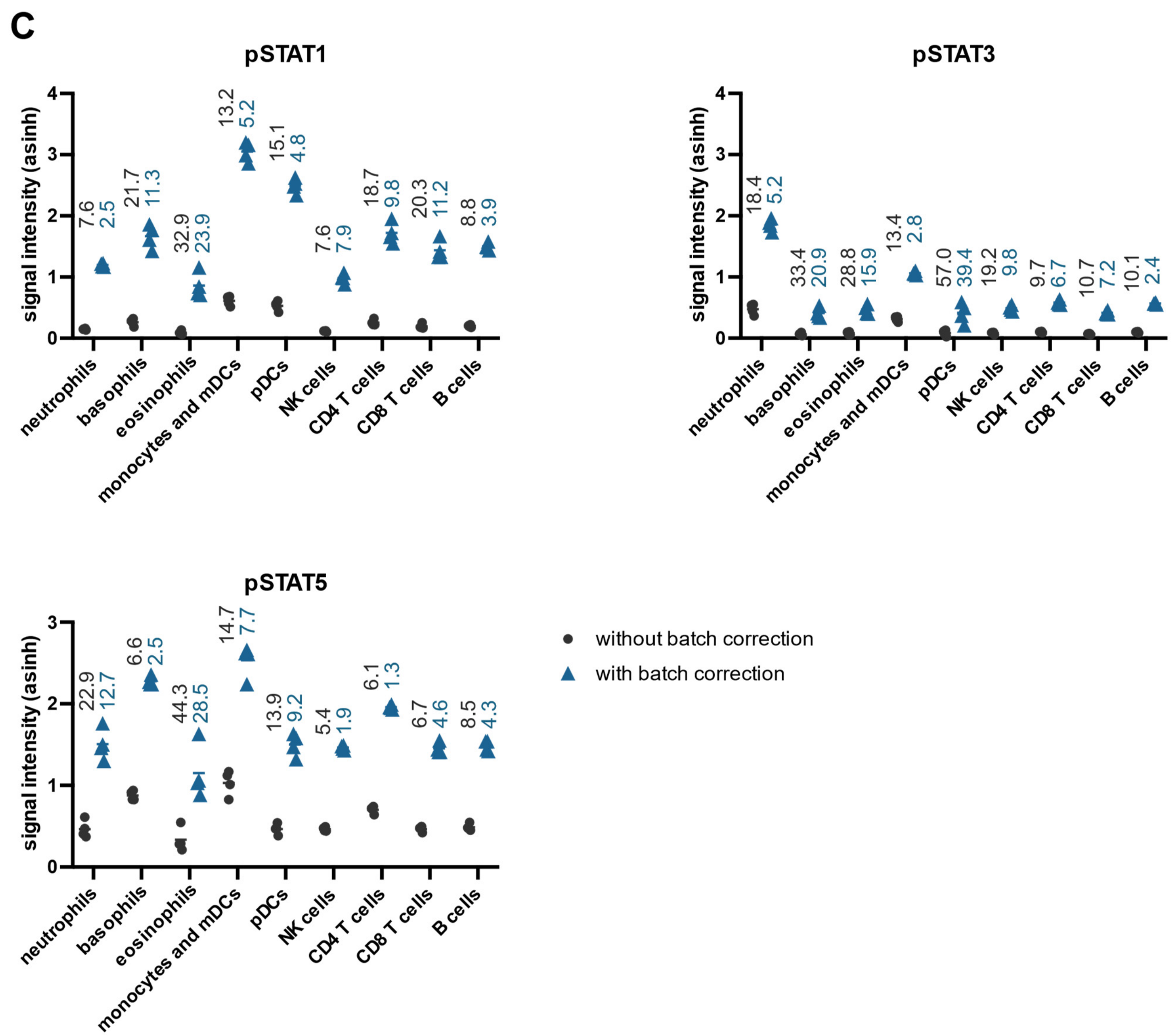

Figure S2: Effects of batch effect correction on cell abundance, surface marker and phospho-protein levels of reference samples (part 2/2). Frequencies **(A)**, lineage marker expression **(B)**, and pSTAT1, 3 and 5 levels **(C)** of major immune cell subsets in the references samples included in each study day were assessed before and after batch correction. Numbers represent coefficients of variation (CV). One dot represents one reference sample. While absolute intensity values changed, no

significant impact of batch correction on group differences between SLE and healthy controls was found (see Figure S3).

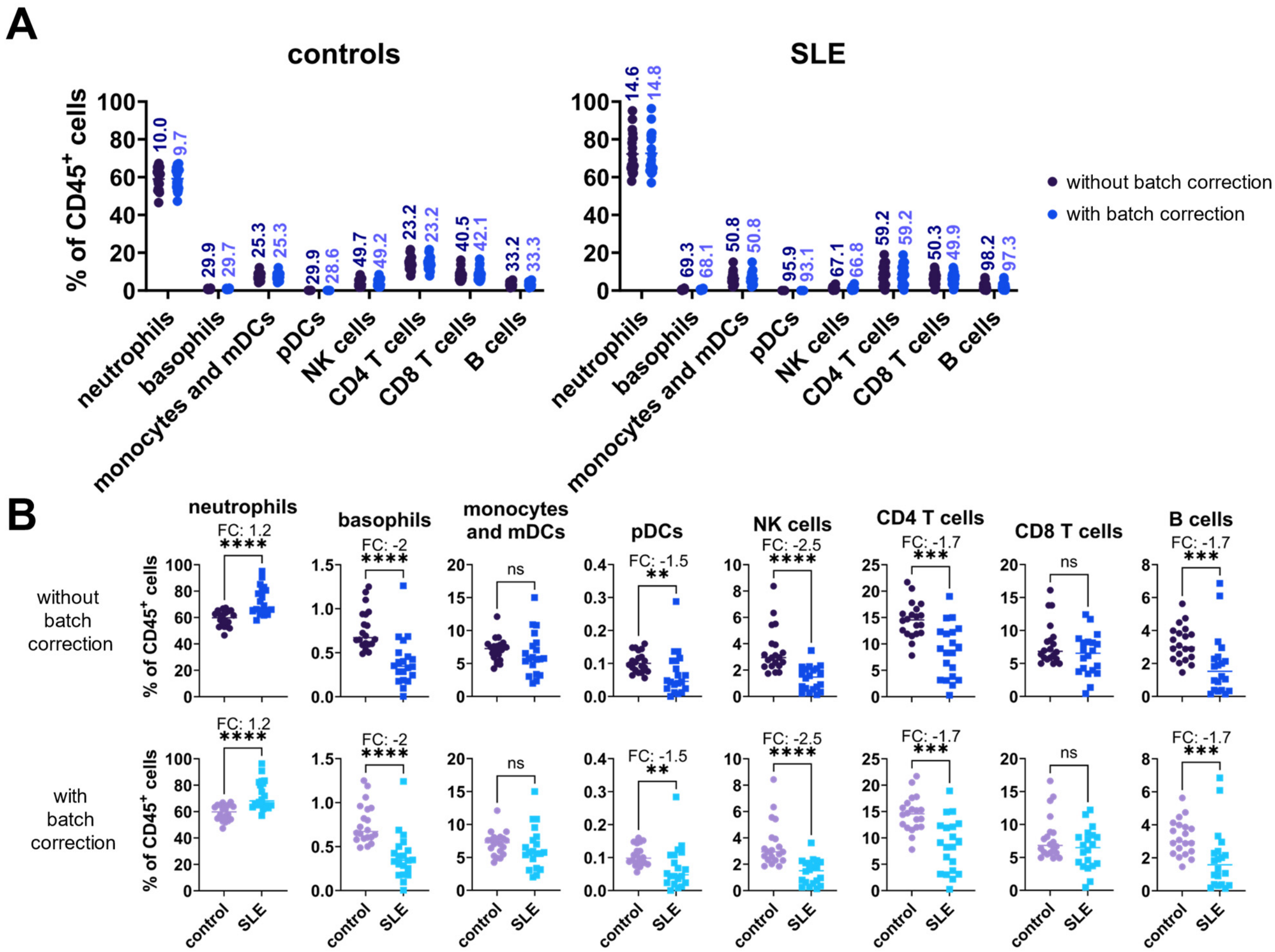

Figure S3: Effects of batch effect correction on cell abundances and pSTAT levels in control and SLE samples (part 1/2). To ensure that batch correction did not impact donor variation and group differences in our study cohort, frequencies of major immune cell subsets and their pSTAT1, 3, and 5 levels were analyzed, and coefficients of variation (CV) were calculated for non-corrected and corrected samples. Group differences in cell abundance between SLE and matched controls were assessed before and after batch correction, using Mann Whitney testing (*, p < 0.05, **, p < 0.01, ***, p < 0.001, ****, p < 0.0001) and fold changes (FC) between healthy controls and SLE patients were calculated. One dot represents one donor. **(A, B)** CV and fold changes between controls and SLE for cell frequencies as percentage of total leukocytes. **(C, D)** CV and fold changes between

controls and SLE as arcsinh (5)-transformed mean signal intensity values. No significant impact of batch correction on group differences between SLE and healthy controls was found.

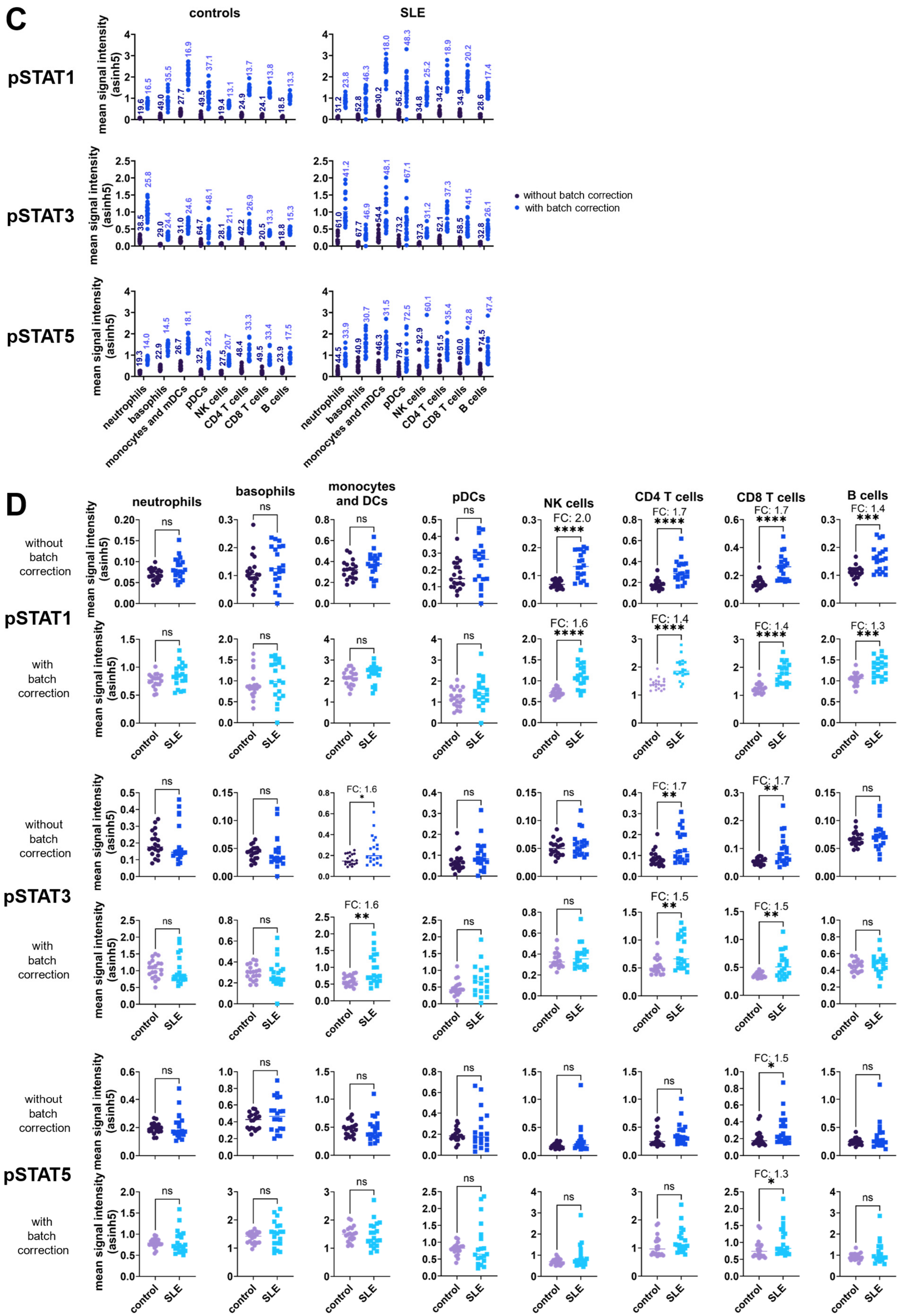
C
controls
SLE
pSTAT1
pSTAT3
pSTAT5
mean signal intensity (asinh5)
without batch correction
with batch correction
neutrophils
basophils
monocytes and mDCs
pDCs
NK cells
CD4 T cells
CD8 T cells
B cells
D
neutrophils
basophils
monocytes and DCs
pDCs
NK cells
CD4 T cells
CD8 T cells
B cells
without batch correction
with batch correction
control
SLE
ns
FC: 2.0
FC: 1.7
FC: 1.7
FC: 1.4
FC: 1.6
FC: 1.4
FC: 1.4
FC: 1.3
FC: 1.6
FC: 1.7
FC: 1.7
FC: 1.6
FC: 1.5
FC: 1.5
FC: 1.5
FC: 1.3

Figure S3: Effects of batch effect correction on cell abundances and pSTAT levels in control and SLE samples (part 2/2). To ensure that batch correction did not impact donor variation and group differences in our study cohort, frequencies of major immune cell subsets and their pSTAT1, 3, and 5 levels were analyzed, and coefficients of variation (CV) were calculated for non-corrected and corrected samples. Group differences in cell abundance between SLE and matched controls were assessed before and after batch correction, using Mann Whitney testing (*, $p < 0.05$, **, $p < 0.01$, ***, $p < 0.001$, ****, $p < 0.0001$) and fold changes (FC) between healthy controls and SLE patients were calculated. One dot represents one donor. **(A, B)** CV and fold changes between controls and SLE for cell frequencies as percentage of total leukocytes. **(C, D)** CV and fold changes between controls and SLE as arcsinh (5)-transformed mean signal intensity values. No significant impact of batch correction on group differences between SLE and healthy controls was found.

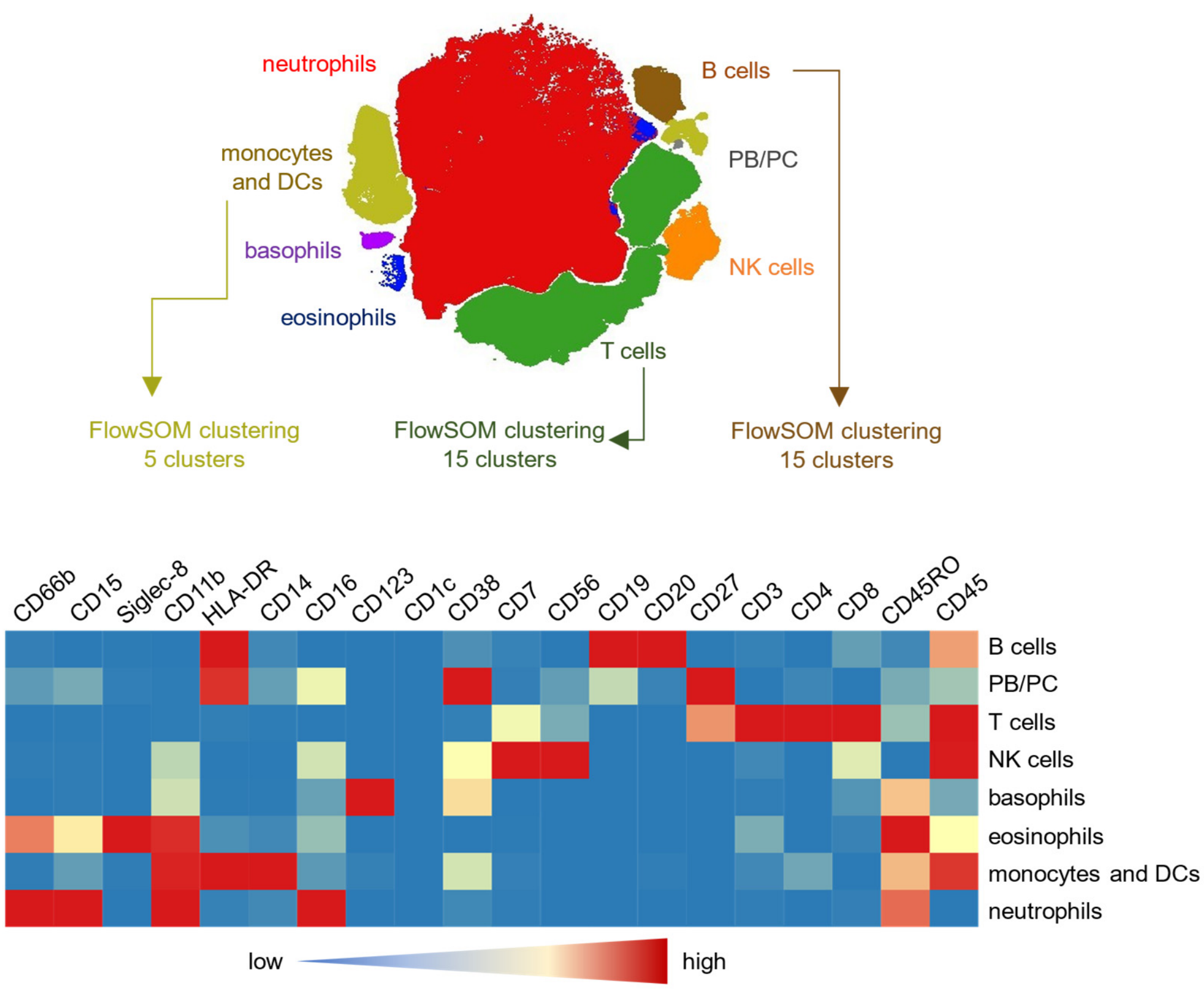

Figure S4: t-SNE map of major leukocyte lineages. Major immune cell subsets in peripheral blood were identified according to the expression of lineage-defining markers and gated in the t-SNE plot. Heatmap showing marker expression profiles of major leukocyte subsets depicted in the map (blue: low signal intensity, red: high signal intensity*)*. CD66b and 16 were excluded from dimension reduction due to batch effects but are displayed in the heatmap to confirm their expression and hence the cell type identities. Concatenated data of 20 healthy controls is shown. Subsequently, monocytes

and DCs, B cells, and T cells were subjected to FlowSOM clustering to identify minor immune cell subsets in those lineages (Table ST1, Figure S4).

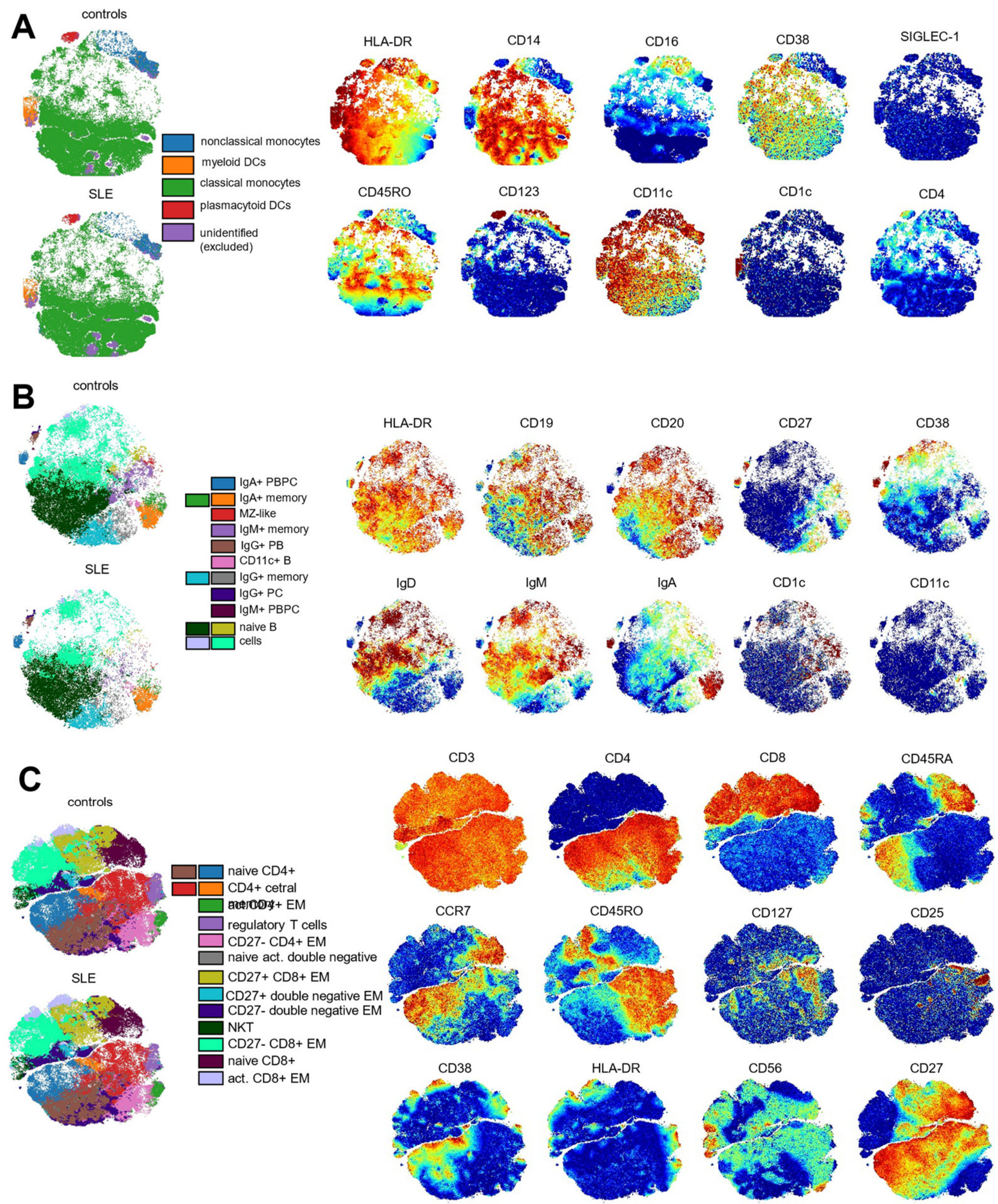

Figure S5: Annotation of monocyte, dendritic cell (DC), B cell and T cell clusters. Monocytes, DCs **(A)**, B cells **(B)** and T cells **(C)** were gated from the global t-SNE map in Figure S2 and clustered by FlowSOM clustering (see Materials & methods for details). FlowSOM clusters were overlaid on the respective t-SNE plot (left side) and annotated according to their cell-surface marker expression profiles. Depicted t SNE plots represent concatenated data of healthy control samples, colored by marker expression (right side; blue: low signal intensity; red: high signal intensity). DC = dendritic cell, PB = plasmablast, PC = plasma cell, EM = effector memory.

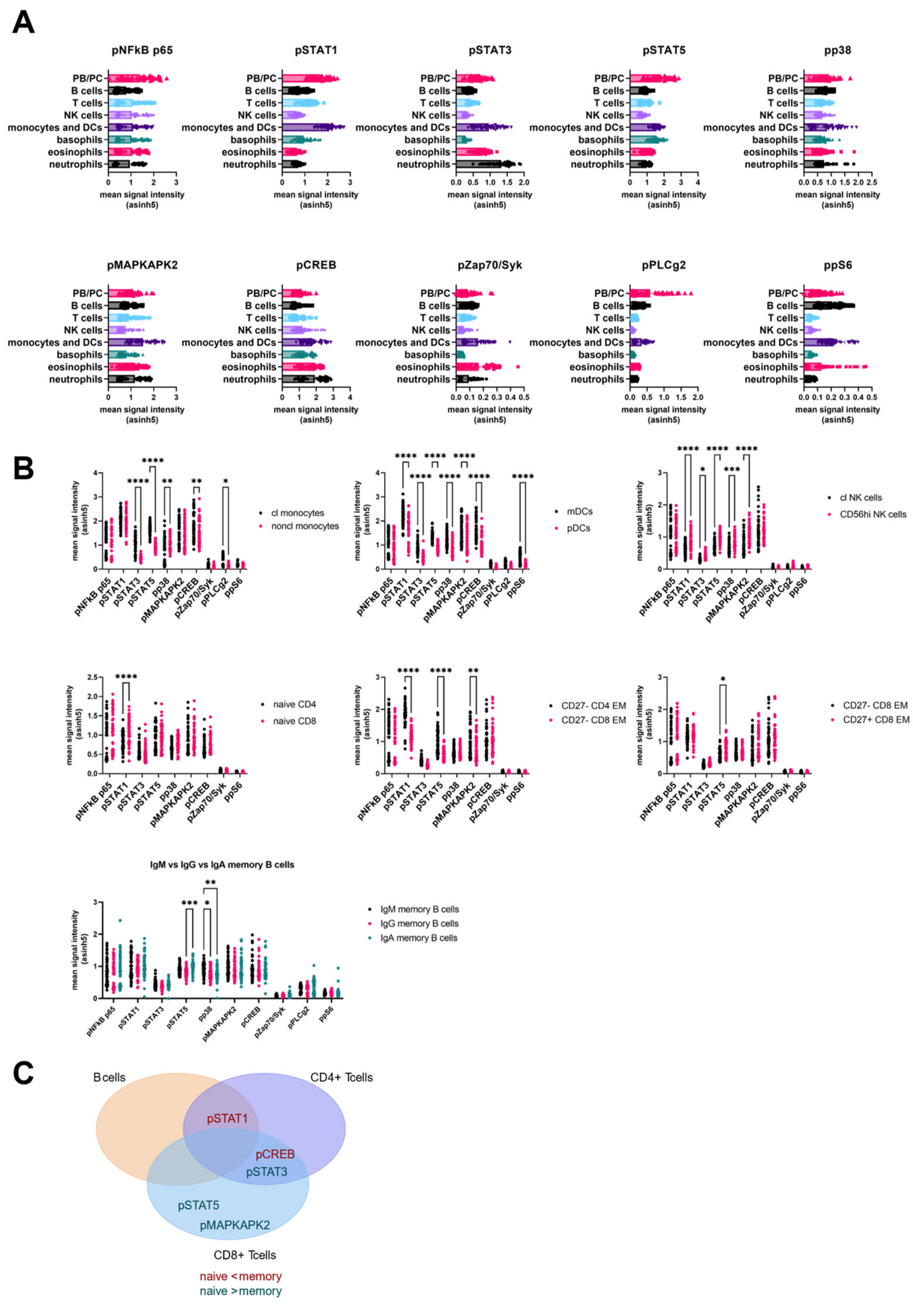

Figure S6: UACT signatures of healthy controls. Major and minor immune cell subsets of healthy control donors were identified using dimension reduction and FlowSOM clustering. (A) Bar and scatter graph showing mean and standard deviation of UACT levels of the ten phosphorylated signal transduction proteins included in this study. Each dot represents one donor. (B) Subsets of monocytes, dendritic cells, NK, T and B cells were compared with regards to their UACT signaling activity. Statistical testing was performed using by two-way ANOVA using Šidak's correction for multiple testing (adjusted p-values, *, p < 0.05; **, p < 0.01; ***, p < 0.001; ****, p < 0.0001). (C)

Venn Diagram depicting the UACT phosphorylation patterns in naive vs. memory compartments of B cells, CD4 and CD8 T cells. Red color indicates lower and green indicates higher phospho-protein levels in naive vs. memory lymphocytes. Only statistically significant differences, as determined by ANOVA, are shown.

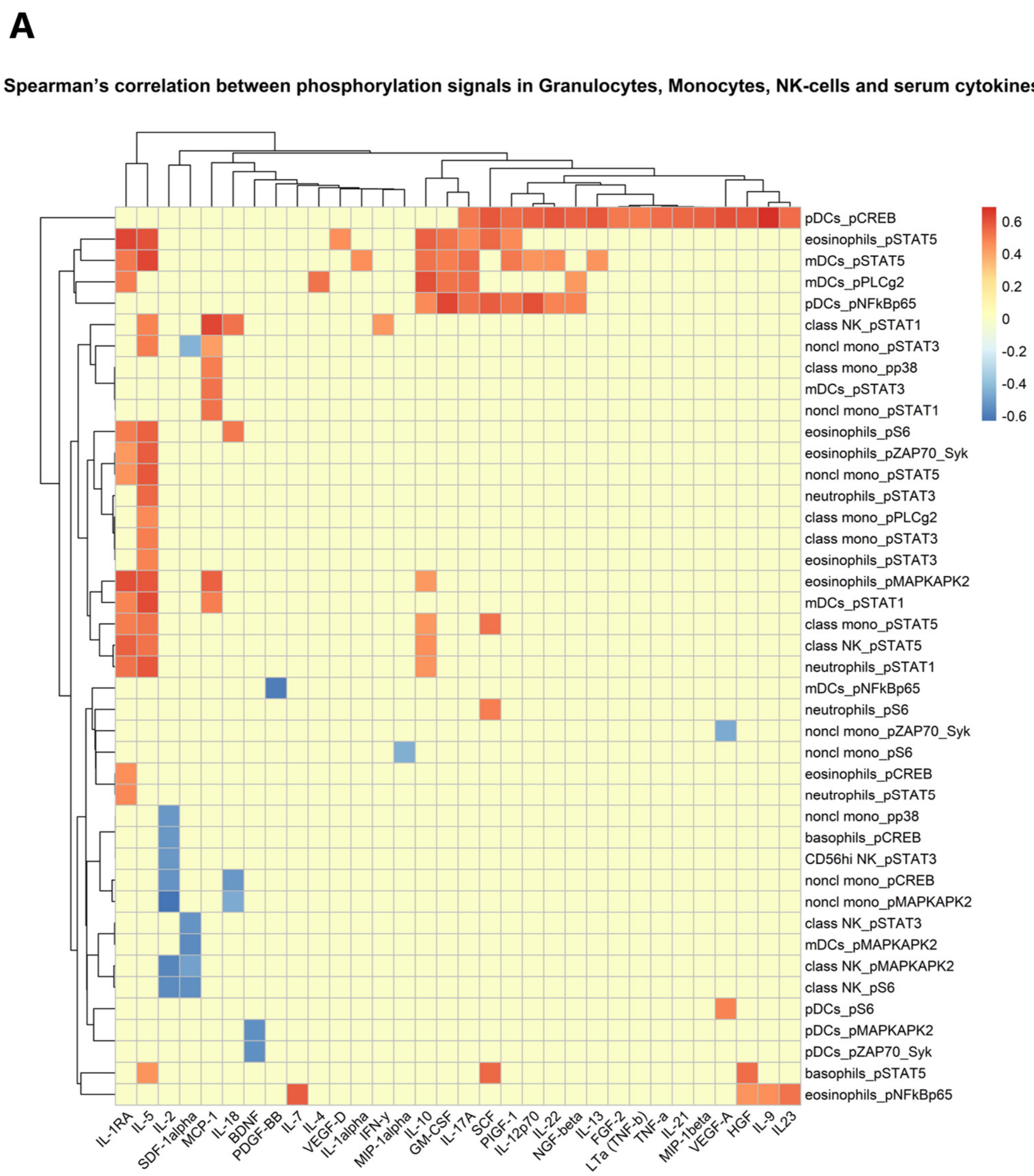

Figure S7: Correlation of phospho-signals with serum cytokines (part 1/3). Signal intensity data from serum cytokine measurements by Luminex and phosphorylated proteins from mass cytometry of innate immune cells (A), and T and B lymphocytes (B,C) were correlated using Spearman's correlation. The statistical significance was established by bootstrap hypothesis testing. Yellow fields represent no correlation, red and blue indicate statistically significant positive (red) or negative (blue)

correlations (non-adjusted p-values, p<0.05). Numbers on the color scale indicate the correlation coefficient.

B

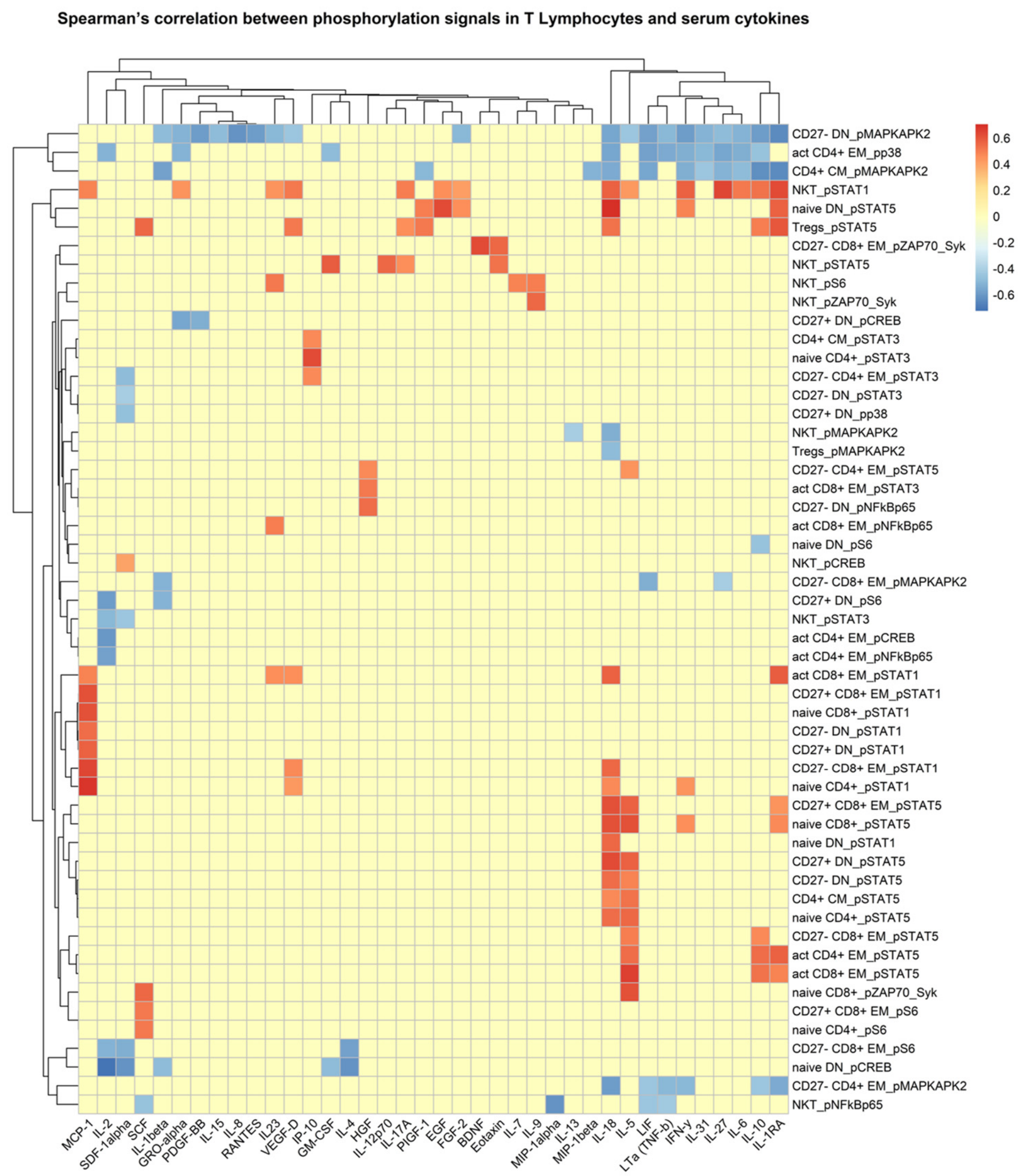

Figure S7: Correlation of phospho-signals with serum cytokines (part 2/3). Signal intensity data from serum cytokine measurements by Luminex and phosphorylated proteins from mass cytometry of innate immune cells (A), and T and B lymphocytes (B,C) were correlated using Spearman's correlation. The statistical significance was established by bootstrap hypothesis testing. Yellow fields represent no correlation, red and blue indicate statistically significant positive (red) or negative (blue)

correlations (non-adjusted p-values, p<0.05). Numbers on the color scale indicate the correlation coefficient.

C

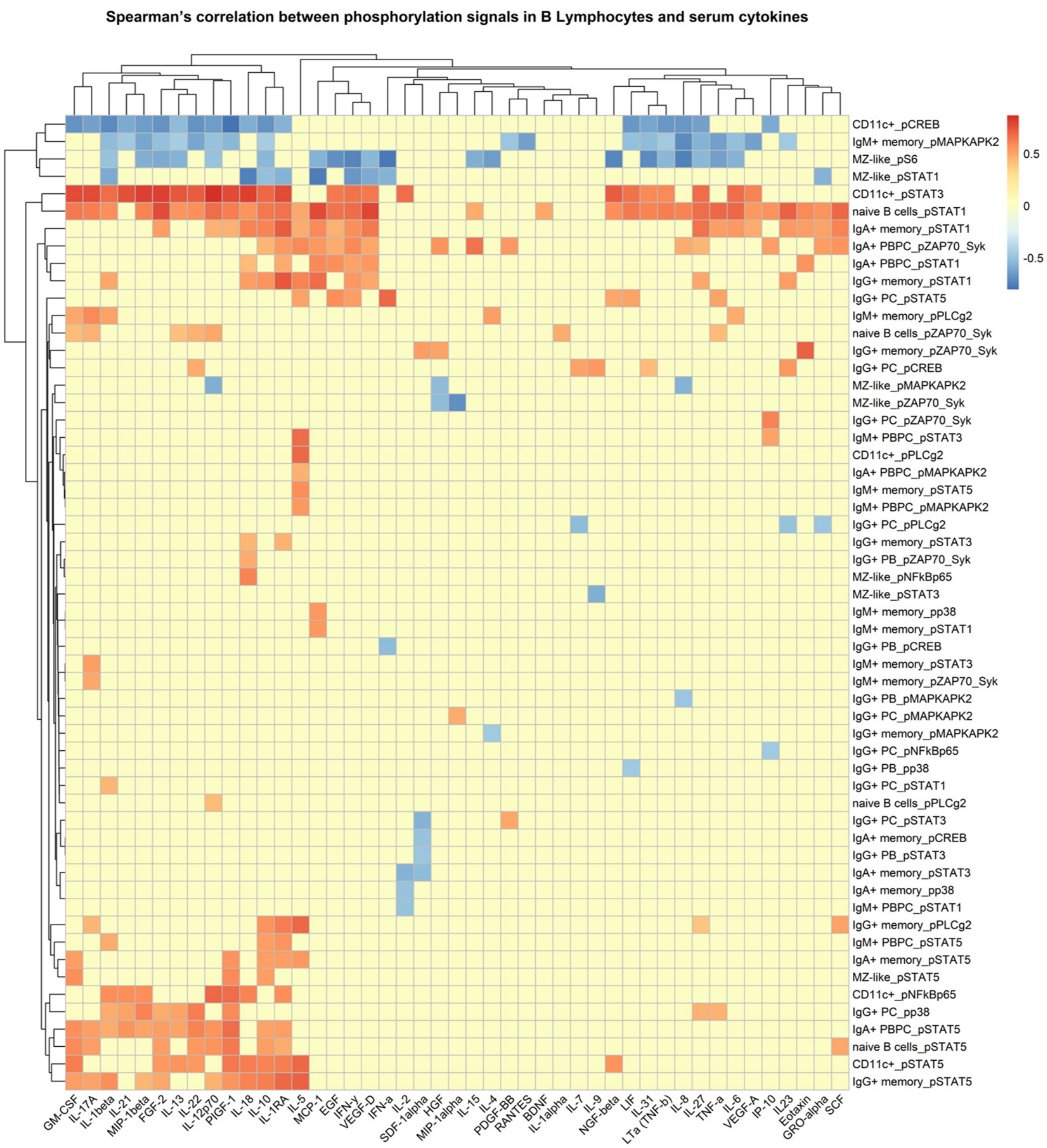

Figure S7: Correlation of phospho-signals with serum cytokines (part 3/3). Signal intensity data from serum cytokine measurements by Luminex and phosphorylated proteins from mass cytometry of innate immune cells (A), and T and B lymphocytes (B,C) were correlated using Spearman's correlation. The statistical significance was established by bootstrap hypothesis testing. Yellow fields

represent no correlation, red and blue indicate statistically significant positive (red) or negative (blue) correlations (non-adjusted p-values, p<0.05). Numbers on the color scale indicate the correlation coefficient.

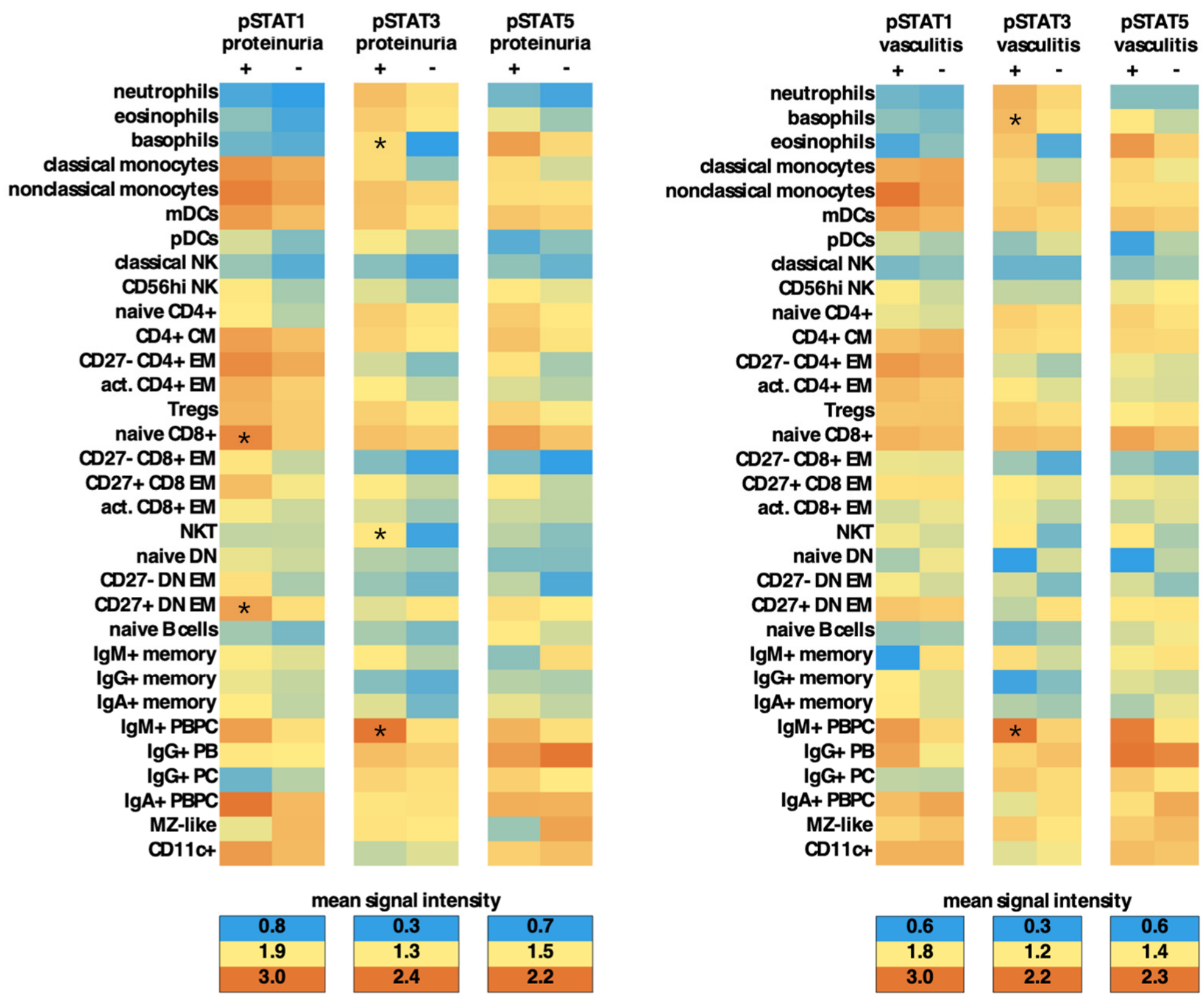

Figure S8: Association of pSTAT UACT signals with proteinuria and vasculitis. SLE patients with proteinuria (n=5, left) *and* vasculitis (n=3, right), were analyzed for *UACT* of STAT1,3,5 proteins compared to patients without these features (*, adjusted p < 0.05). Mean signal intensities across each group in each leukocyte subset are represented.

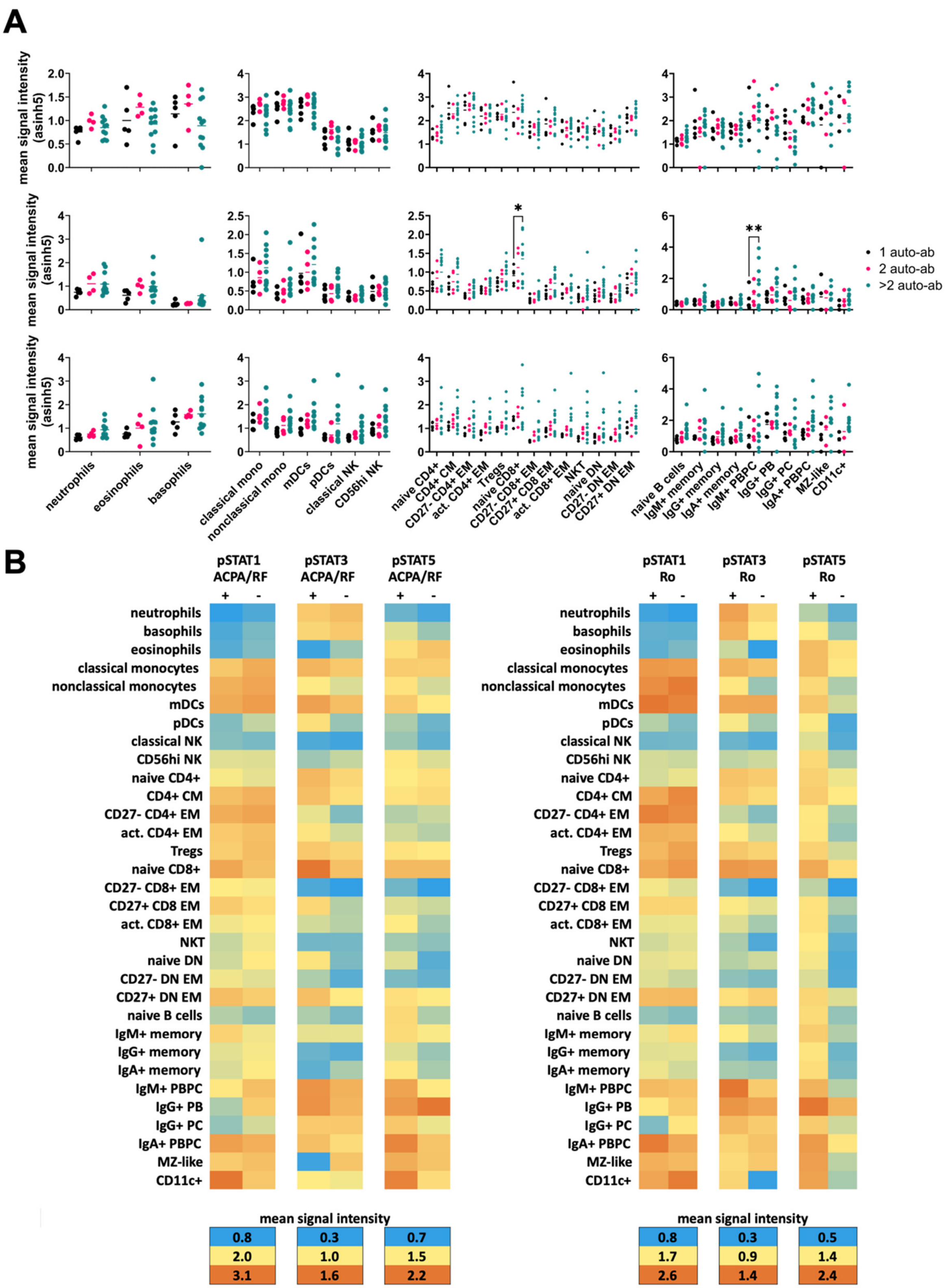
A
mean signal intensity (asinh5)
1 auto-ab
2 auto-ab
>2 auto-ab
*
**
neutrophils
eosinophils
basophils
classical mono
nonclassical mono
mDCs
pDCs
classical NK
CD56hi NK
naive CD4+
CD4+ CM
CD27- CD4+ EM
act. CD4+ EM
Tregs
naive CD8+
CD27- CD8+ EM
CD27+ CD8 EM
act. CD8+ EM
NKT
naive DN
CD27- DN EM
CD27+ DN EM
naive B cells
IgM+ memory
IgG+ memory
IgA+ memory
IgM+ PBPC
IgG+ PB
IgG+ PC
IgA+ PBPC
MZ-like
CD11c+
B
pSTAT1 ACPA/RF
pSTAT3 ACPA/RF
pSTAT5 ACPA/RF
pSTAT1 Ro
pSTAT3 Ro
pSTAT5 Ro
+ -
neutrophils
basophils
eosinophils
classical monocytes
nonclassical monocytes
mDCs
pDCs
classical NK
CD56hi NK
naive CD4+
CD4+ CM
CD27- CD4+ EM
act. CD4+ EM
Tregs
naive CD8+
CD27- CD8+ EM
CD27+ CD8 EM
act. CD8+ EM
NKT
naive DN
CD27- DN EM
CD27+ DN EM
naive B cells
IgM+ memory
IgG+ memory
IgA+ memory
IgM+ PBPC
IgG+ PB
IgG+ PC
IgA+ PBPC
MZ-like
CD11c+
mean signal intensity
0.8 0.3 0.7
2.0 1.0 1.5
3.1 1.6 2.2
mean signal intensity
0.8 0.3 0.5
1.7 0.9 1.4
2.6 1.4 2.4

Figure S9: Association of pSTAT signals with the presence of different autoantibodies. (A) SLE patients were grouped according to the number of autoantibody specificities (one (anti-dsDNA autoantibodies/nucleosome only, black), two (pink) or more than two (green)) found in their serum and analyzed for differential STAT1,3,5 UACT across all blood immune cell subsets. arcsinh-transformed mean signal intensity values using a scale argument of five are plotted. Line indicates mean. Changes in STAT phosphorylation between groups were determined by two-way ANOVA with Tukey's correction for multiple testing (*, adjusted $p < 0.05$). **(B)** SLE patients positive for anti-citrillunated peptide antibodies (ACPA) and/or rheumatoid factor (RF) (n=7) and patients with anti-Ro antibodies (n=13) were analyzed for UACT of STAT1,3,5 proteins compared to patients without these features. Mean signal intensities across each group in each leukocyte subset are represented.

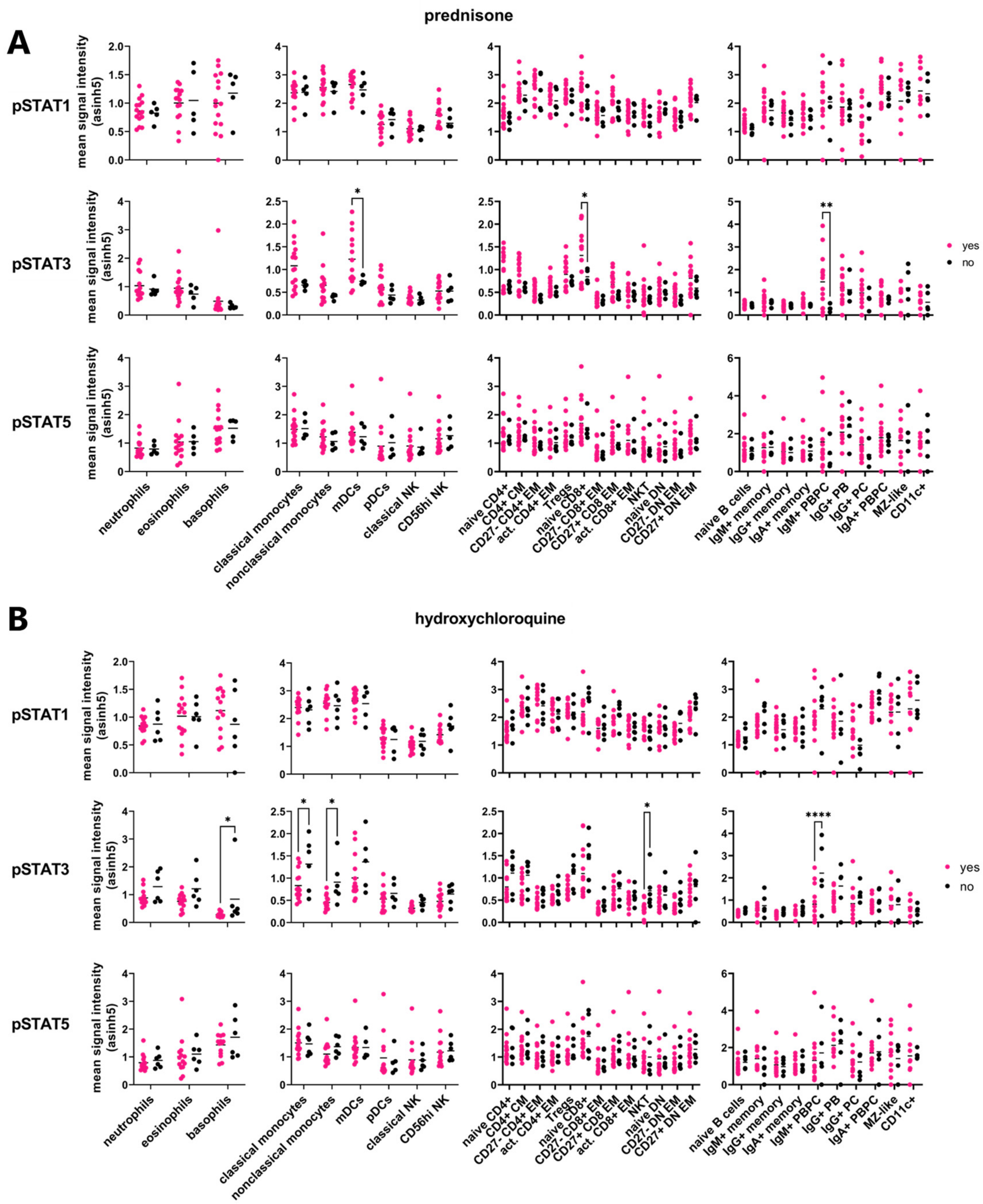

Figure S10: Influence of immunomodulatory medication on the pSTAT levels (part 1/3). Arcsinh-transformed mean signal intensity values of pSTAT1, 3 and 5 were compared between patients treated (pink, “yes”) or not treated (black, “no”) with prednisone **(A)**, hydroxychloroquine **(B)**, methotrexate **(C)**, azathioprine **(D)**, and belimumab **(E)**. Statistical testing was performed using

two-way ANOVA with Šidak's correction for multiple testing (*, p < 0.05; **, p < 0.01; ****, p < 0.0001).

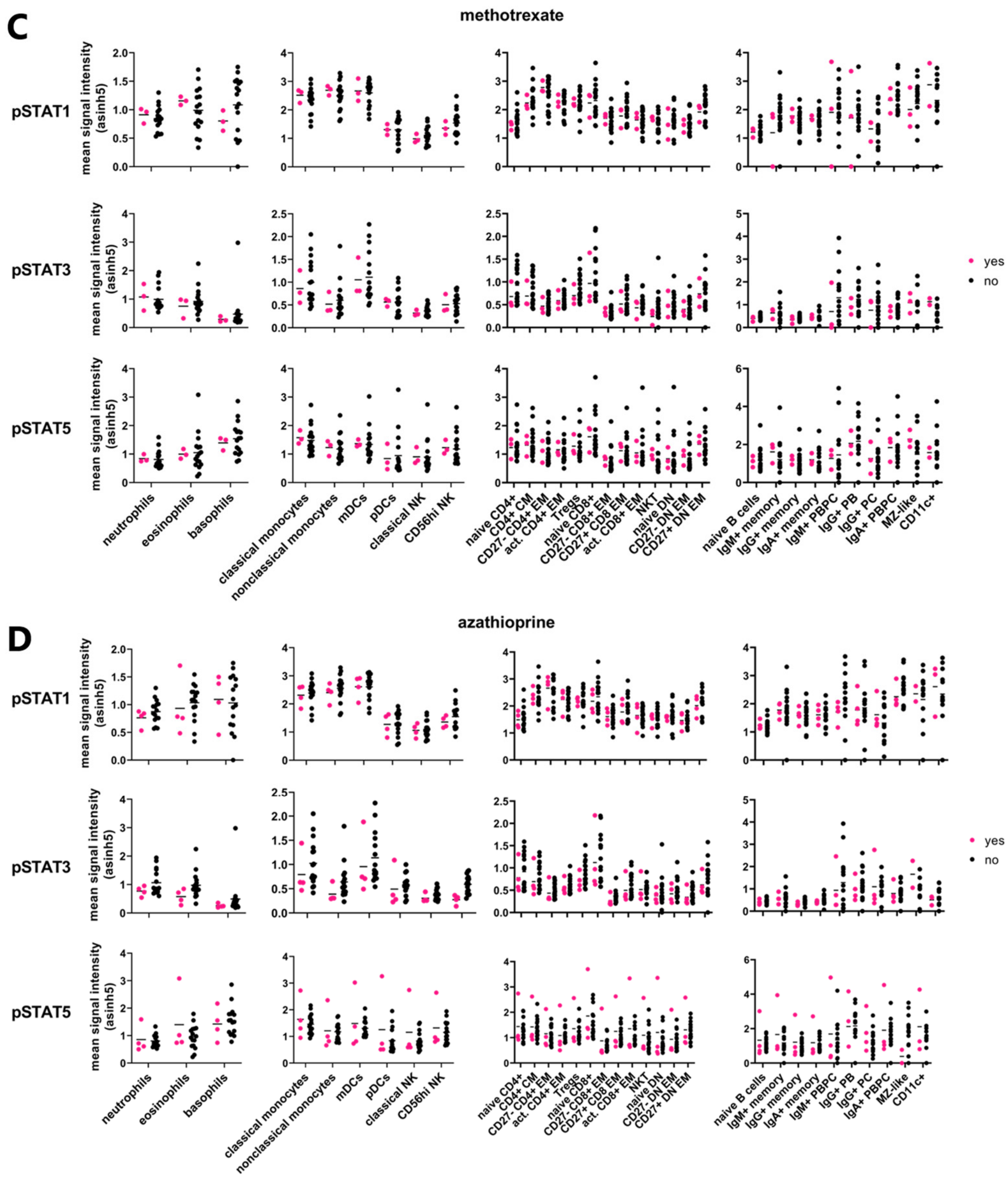

Figure S10: Influence of immunomodulatory medication on the pSTAT levels (part 2/3). Arcsinh-transformed mean signal intensity values of pSTAT1, 3 and 5 were compared between patients treated (pink, "yes") or not treated (black, "no") with prednisone **(A)**, hydroxychloroquine **(B)**, methotrexate **(C)**, azathioprine **(D)**, and belimumab **(E)**. Statistical testing was performed using two-way ANOVA with Šidak's correction for multiple testing (*, p < 0.05; **, p < 0.01; ****, p < 0.0001).

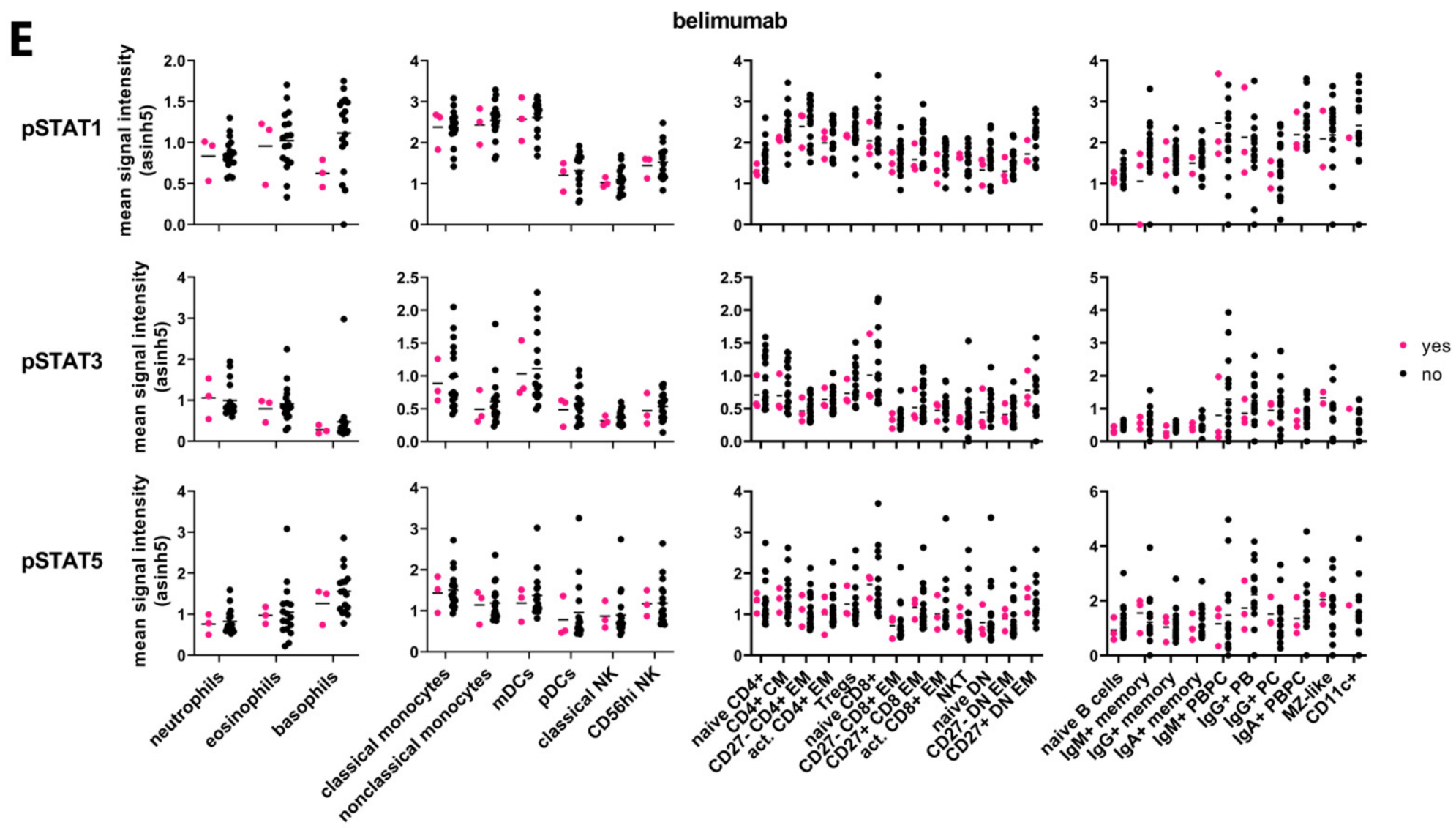

Figure S10: Influence of immunomodulatory medication on the pSTAT levels (part 3/3). Arcsinh-transformed mean signal intensity values of pSTAT1, 3 and 5 were compared between patients treated (pink, “yes”) or not treated (black, “no”) with prednisone **(A)**, hydroxychloroquine **(B)**, methotrexate **(C)**, azathioprine **(D)**, and belimumab **(E)**. Statistical testing was performed using two-way ANOVA with Šidak’s correction for multiple testing (*, $p < 0.05$; **, $p < 0.01$; ****, $p < 0.0001$).

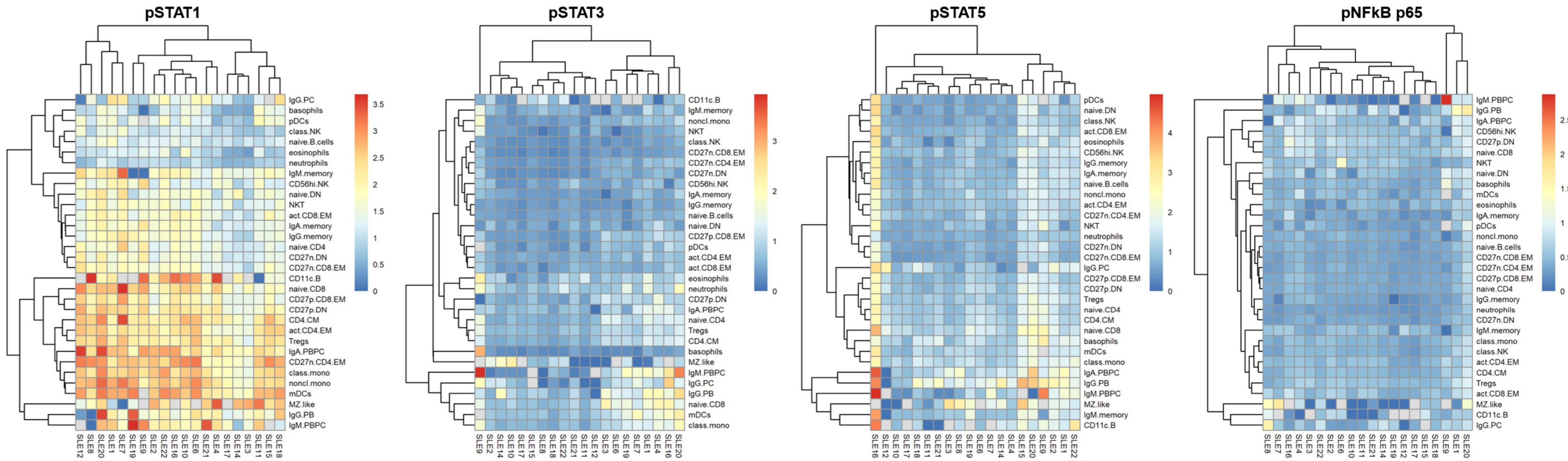

Figure S11: Heatmap representation of mean signal intensities of phosphorylated signal transduction proteins across major and minor immune cells in individual donors (part 1/2). Arcsinh-transformed mean signal intensity data of phosphorylated proteins are shown.

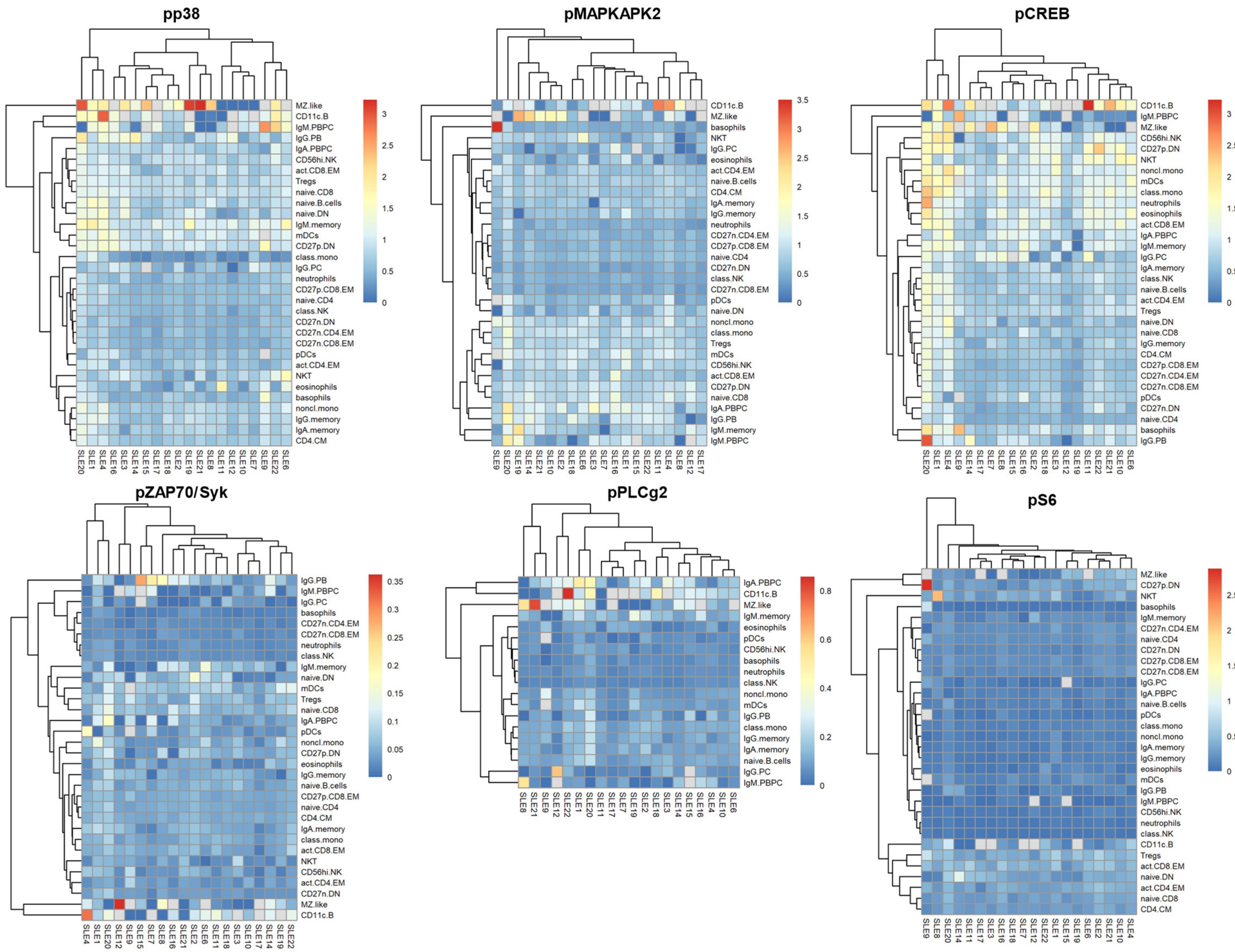

Figure S11: Heatmap representation of mean signal intensities of phosphorylated signal transduction proteins across major and minor immune cells in individual donors (part 2/2). Arcsinh-transformed mean signal intensity data of phosphorylated proteins are shown.

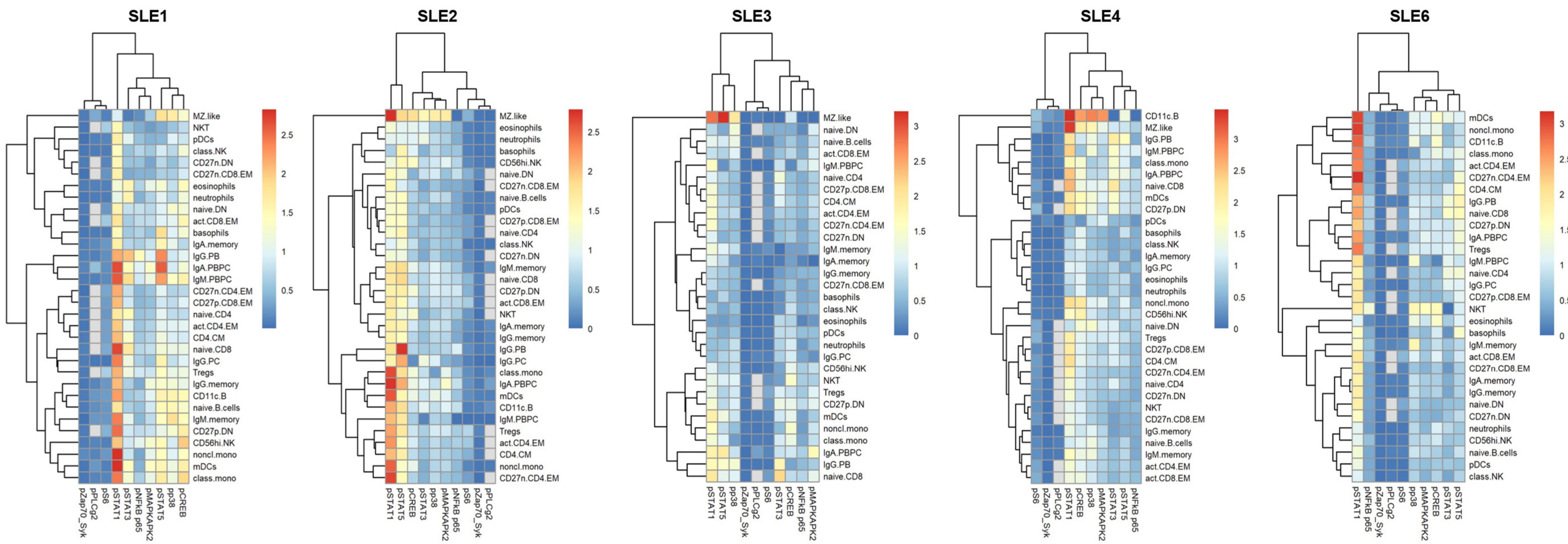

Figure S12: Heatmap representation of individual donors' phospho-signatures (part 1/4). Individual SLE patients were analyzed for arcsinh-transformed mean signal intensities of phospho-proteins across all major and minor immune cell subsets to capture their individual UACT signaling footprint.

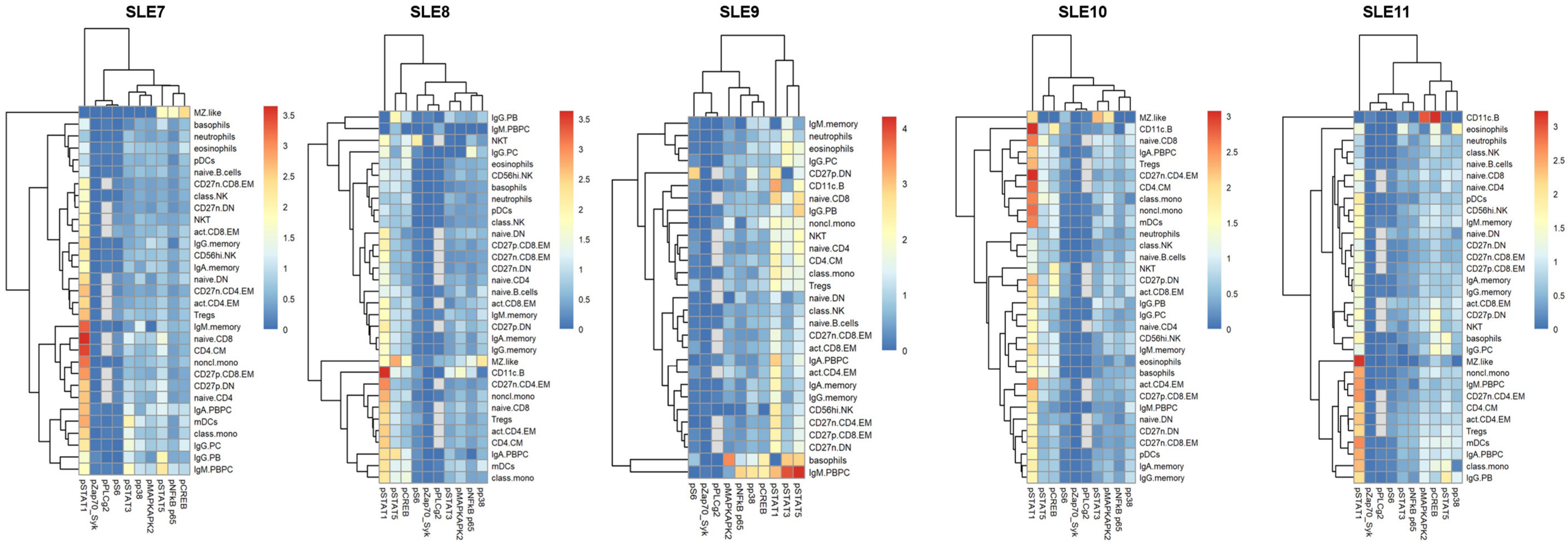

Figure S12: Heatmap representation of individual donors' phospho-signatures (part 2/4). Individual SLE patients were analyzed for arcsinh-transformed mean signal intensities of phospho-proteins across all major and minor immune cell subsets to capture their individual UACT signaling footprint.

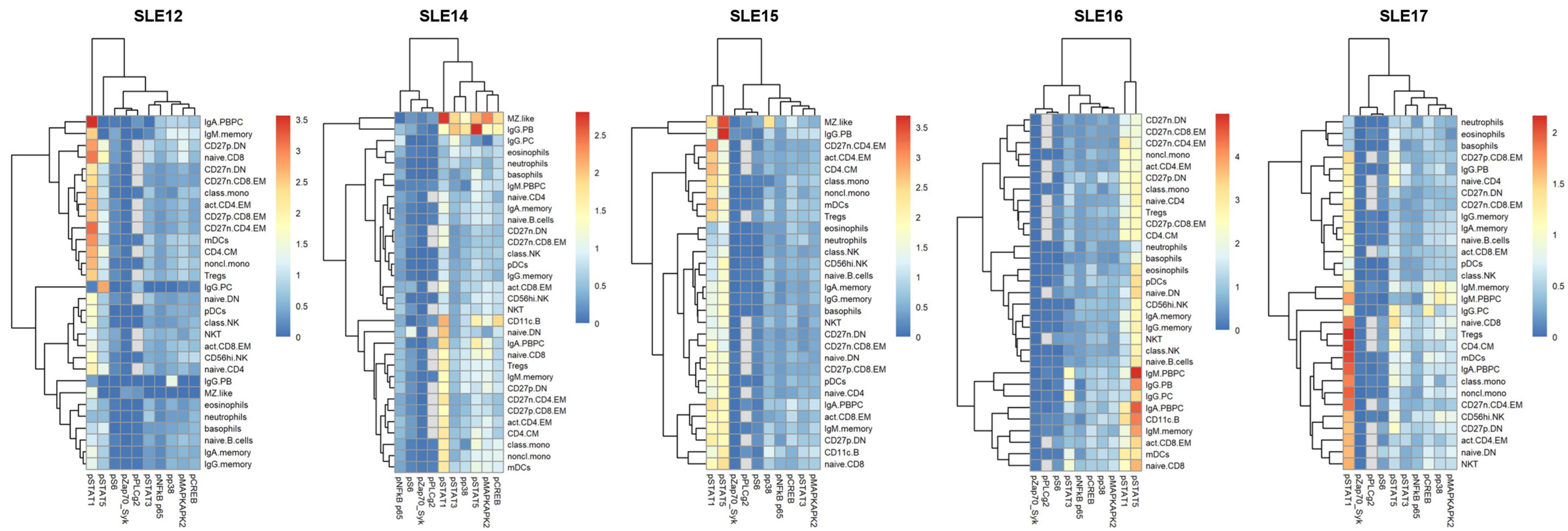

Figure S12: Heatmap representation of individual donors' phospho-signatures (part 3/4). Individual SLE patients were analyzed for arcsinh-transformed mean signal intensities of phospho-proteins across all major and minor immune cell subsets to capture their individual UACT signaling footprint.

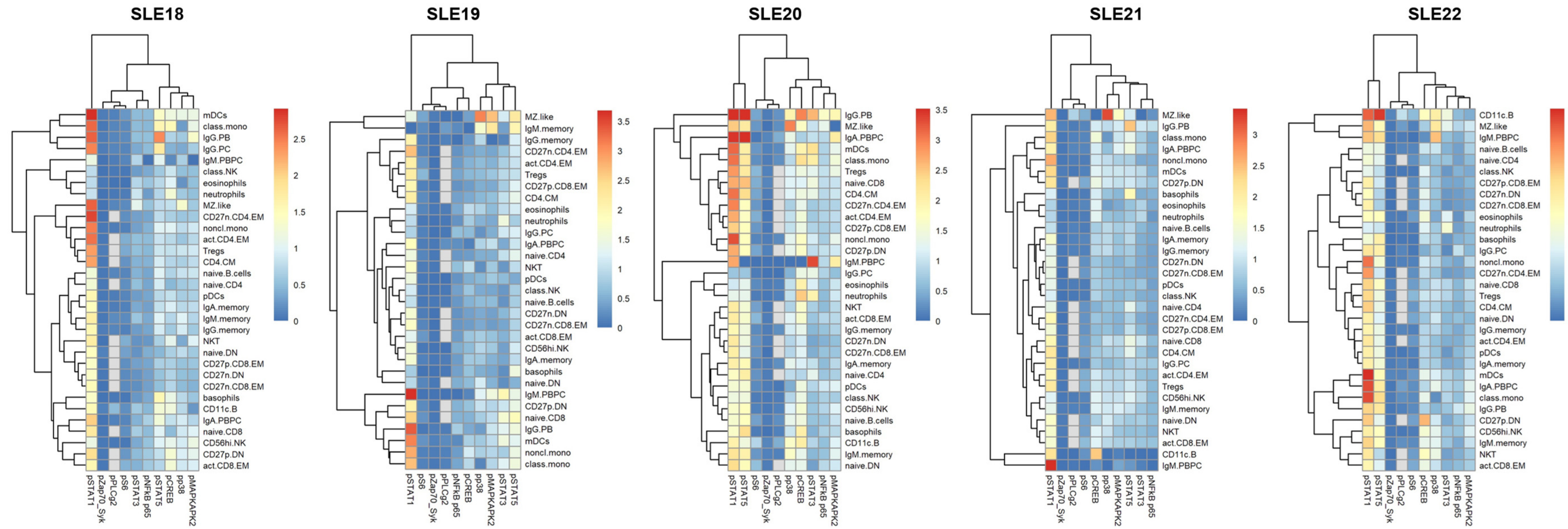

Figure S12: Heatmap representation of individual donors' phospho-signatures (part 4/4). Individual SLE patients were analyzed for arcsinh-transformed mean signal intensities of phospho-proteins across all major and minor immune cell subsets to capture their individual UACT signaling footprint.

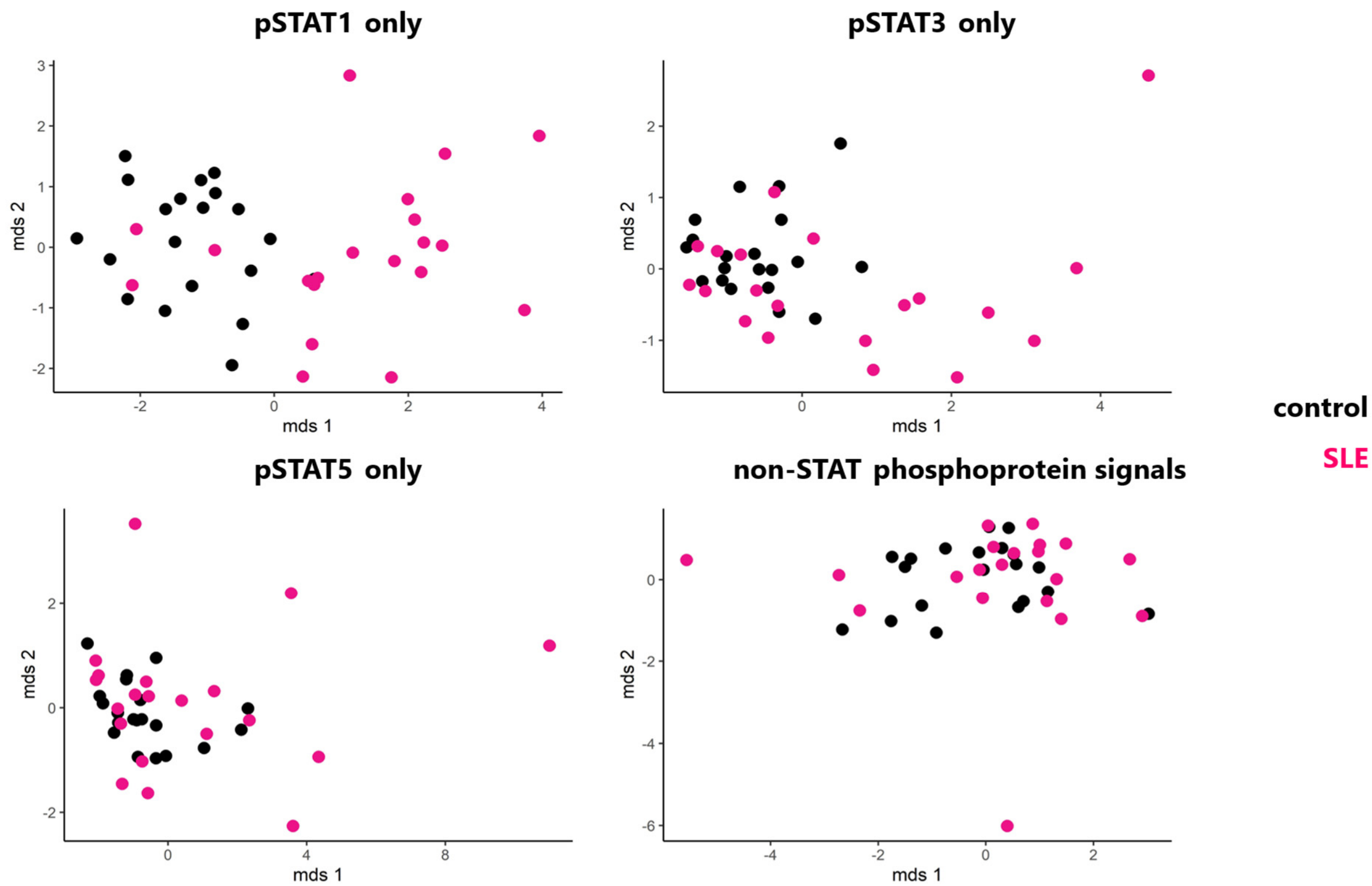

Figure S13: MDS plots of phosphorylation signals across different UACT readouts. pSTAT1,3 and 5 or a combination of pNFκBp65, pp38, pMAPKAPK2, pCREB, pZAP70, pPLCγ2 (non-STAT) mean signal intensity values across all major and minor immune cell subsets were used to calculate dimension reduction by multidimensional scaling (mds) for SLE patients (pink) and controls (black) to investigate the potential of these readouts to distinguish patients and healthy controls.

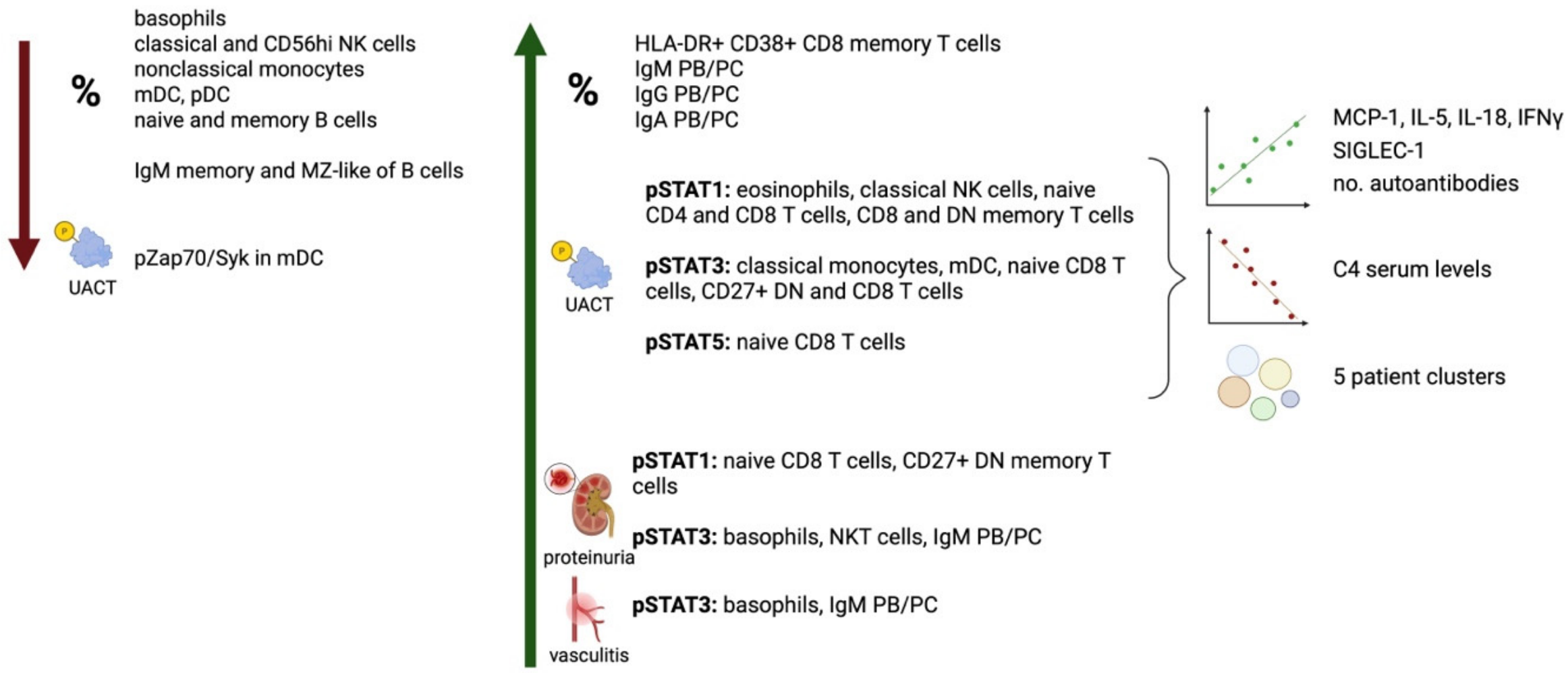

Figure S14: Summary of dysregulated immunological features of SLE and their association with clinical manifestations. Created in BioRender. Burns, M. (2026) https://BioRender.com/dbtehve

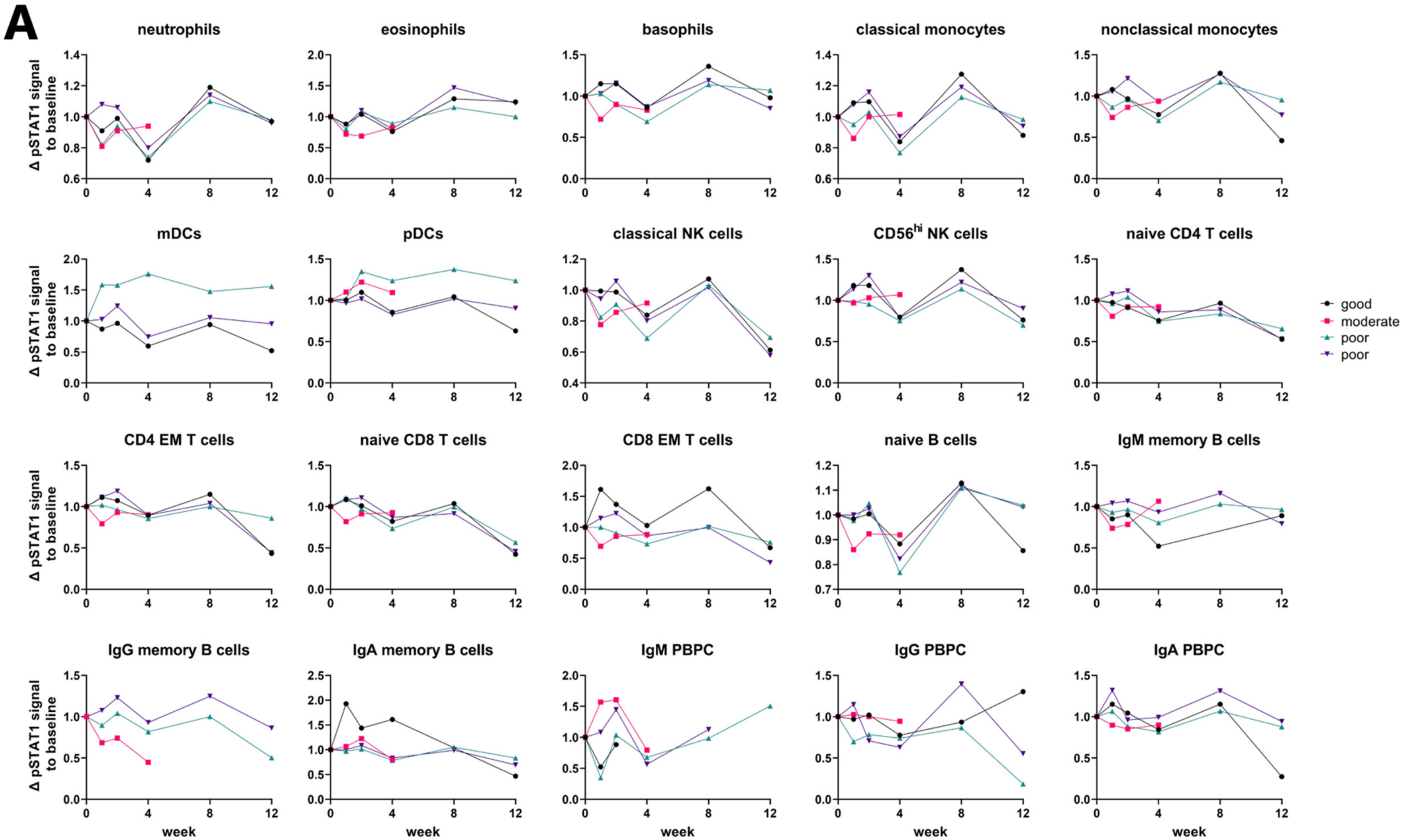

Figure S15: pSTAT1, 3 and 5 kinetics under treatment with baricitinib (part 1/3). Fold changes in STAT1 **(A)**, pSTAT3 **(B)** and pSTAT5 **(C)** levels after initiation of baricitinib treatment are depicted for all donors. Mean signal intensities were analyzed and normalized

to baseline (before treatment initiation). Missing values indicate that the respective subset was not detected at the given time point and signal intensity values could thus not be analyzed.

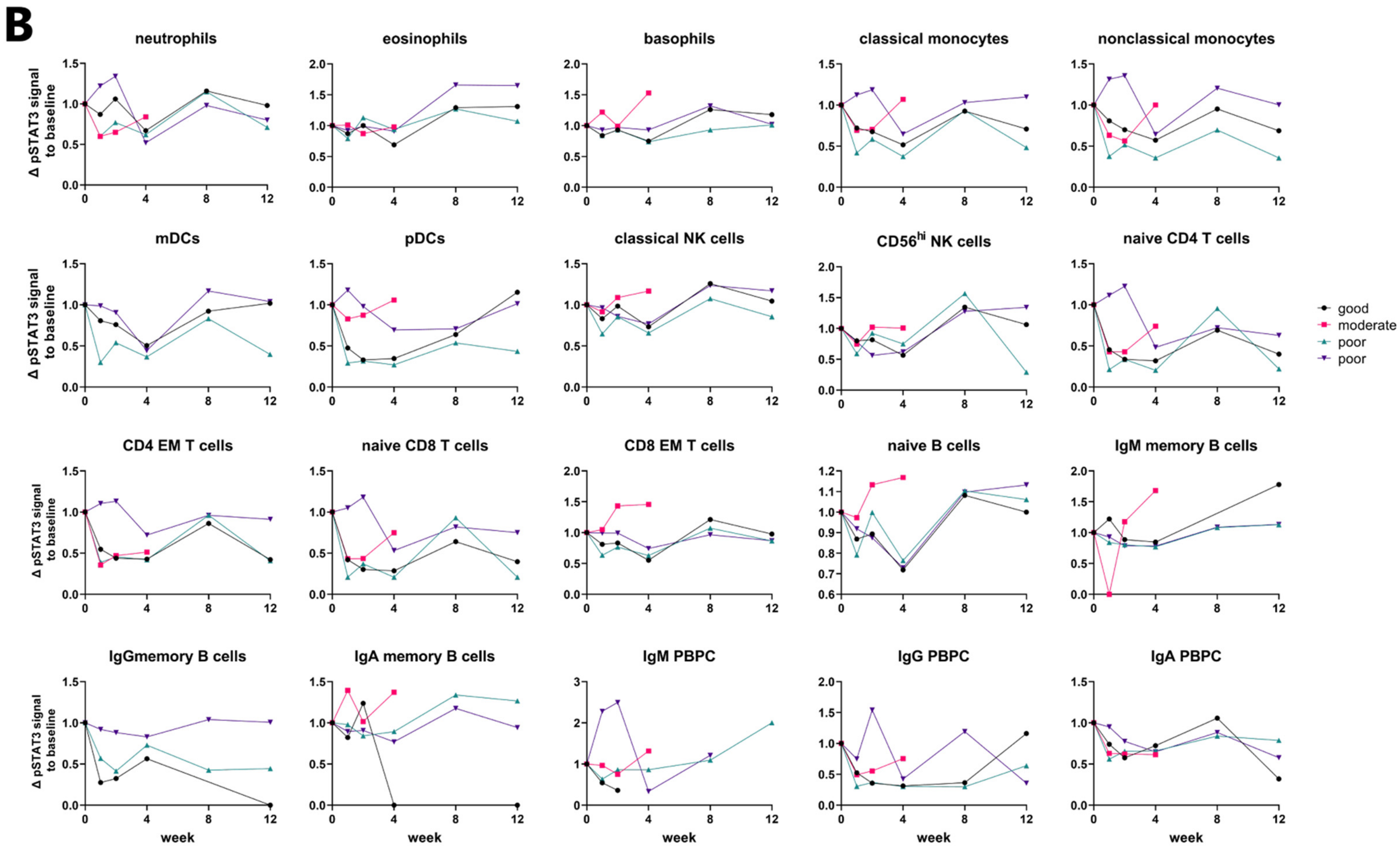

Figure S15: pSTAT1, 3 and 5 kinetics under treatment with baricitinib (part 2/3). Fold changes in STAT1 **(A)**, pSTAT3 **(B)** and pSTAT5 **(C)** levels after initiation of baricitinib treatment are depicted for all donors. Mean signal intensities were analyzed and normalized

to baseline (before treatment initiation). Missing values indicate that the respective subset was not detected at the given time point and signal intensity values could thus not be analyzed.

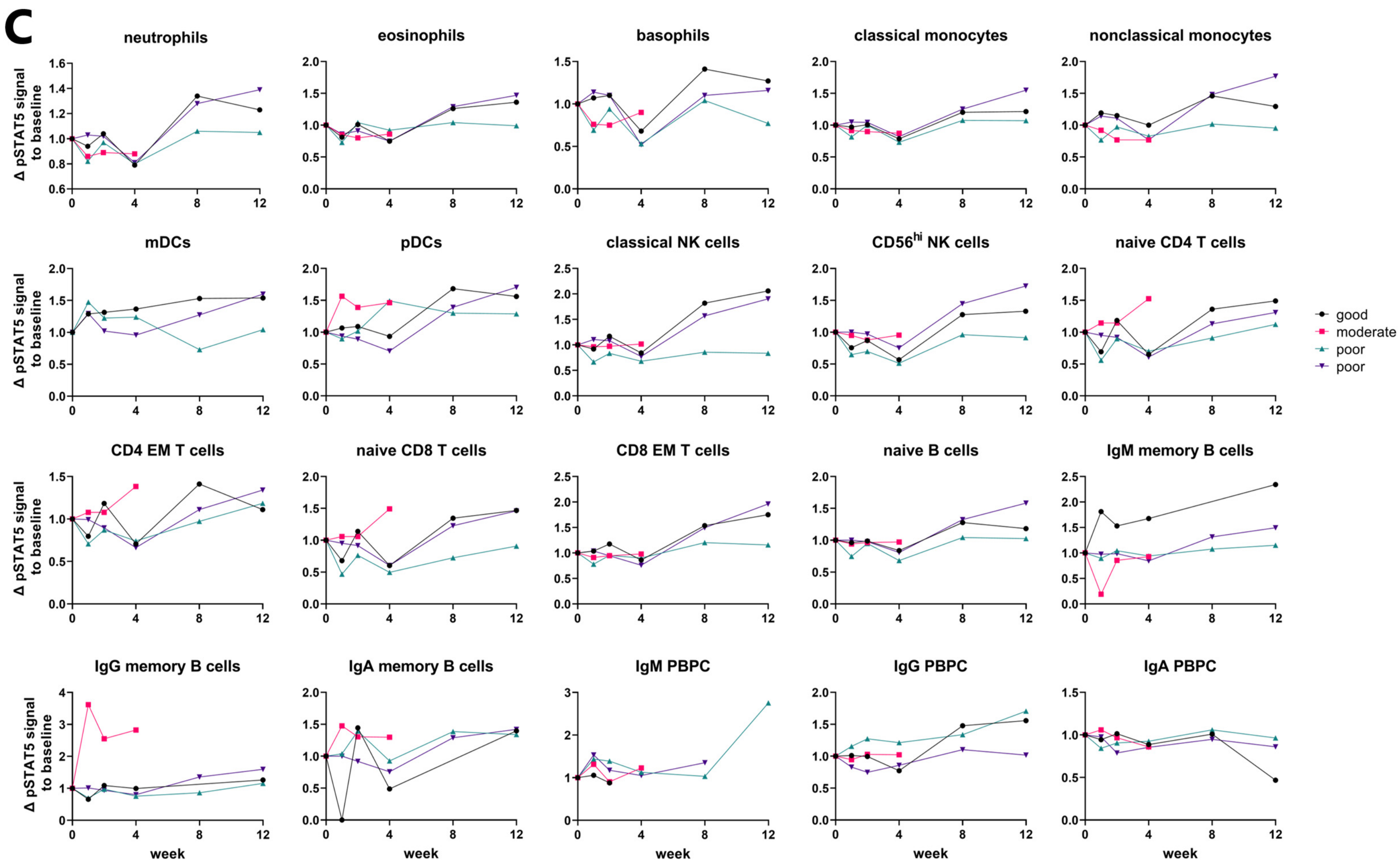

Figure S15: pSTAT1, 3 and 5 kinetics under treatment with baricitinib (part 3/3). Fold changes in STAT1 **(A)**, pSTAT3 **(B)** and pSTAT5 **(C)** levels after initiation of baricitinib treatment are depicted for all donors. Mean signal intensities were analyzed and normalized

to baseline (before treatment initiation). Missing values indicate that the respective subset was not detected at the given time point and signal intensity values could thus not be analyzed.

1.2 Supplementary Tables

Table ST1: Antibody conjugates for mass cytometry and parameters for dimension reduction and clustering analyses.

isotope mass	metal	antibody target or reagent function	antibody clone	supplier	Cat no.	RRID	cell-surface (sf) /intracellular (ic)	used for global fit-SNE	used for monocyte/DC fit-SNE + clustering	used for B/PBPC cell fit-SNE + clustering	used for T cell fit-SNE + clustering
**89**	Y	CD15	W6D3	Biolegend	323002	AB_756008	sf				
**103**	Rh	mDOTA									
**104**	Pd	barcoding									
**105**	Pd	barcoding									
**106**	Pd	barcoding									
**108**	Pd	barcoding									
**110**	Pd	barcoding									
**113**	In	CD66b	REA306	Miltenyi Biotec	130-108-019	AB_2658994	sf				
**115**	In	Siglec 8	#837535	R & D Systems	MAB7975		sf	x			
**140**	Ce	CD14	RMO52	Beckman Coulter	custom order		sf	x	x		
**141**	Pr	CD127	A019D5	Biolegend	351302	AB_10718513	sf				x
**142**	Nd	cleaved caspase 3	D3E9	Fluidigm	3142004A	AB_2847863	ic				
**143**	Nd	CD19	BU12	DRFZ	n.a.		sf	x		x	
**144**	Nd	pPLCg2 (Y759)	K86-689.37	Fluidigm	3144015A	AB_2661830	ic				

**145**	Nd	CD4	RPAT4	Biolegend	300502	AB_314070	sf	x	x		x
**146**	Nd	CD45RO	UCHL1	DRFZ	n.a.		sf		x		x
**147**	Sm	CD20	Rituximab	Charité	n.a.		sf	x		x	
**148**	Nd	IgA	IS11-8E10	Miltenyi Biotec	130-093-073	AB_1036150	sf			x	
**149**	Sm	Syk	4D10.2	Fluidigm	3149020B		ic			x	
**150**	Nd	pSTAT5 (Y694)	47	Fluidigm	3150005A	AB_2744690	ic				
**151**	Eu	CD123	6H6	Biolegend	306002	AB_314576	sf	x	x		
**152**	Sm	CD45RA	4G11	DRFZ	n.a.		sf				x
**153**	Eu	pSTAT1 (Y701)	4a	Fluidigm	3153005A	AB_2744689	ic				
**154**	Sm	CD1c	AT5-8E7	Miltenyi Biotec	130-108-032	AB_2661165	sf	x	x	x	
**155**	Gd	CD27	2E4	Sanquin	M9185		sf			x	x
**156**	Gd	p38 (T180/182)	D3F9	Fluidigm	3156002A	AB_2661826	ic				
**158**	Gd	pSTAT3 (Y705)	4/p-STAT3	Fluidigm	3158005A	AB_2811100	ic				
**159**	Tb	pMAPKAPK2(T334)	27B7	Fluidigm	3159010A	AB_2661828	ic				
**160**	Gd	CD11c	BU15	Biolegend	337202	AB_1236381	sf			x	
**161**	Dy	CD7	CD7-6B7	Biolegend	343102	AB_1659214	sf	x			x
**162**	Dy	IgM	MHM-88	Biolegend	314502	AB_493003	sf			x	
**163**	Dy	CD197	G043H7	Biolegend	353202	AB_10945157	sf				x
**165**	Ho	pCREB (S133)	87G3	Fluidigm	3165009A	AB_2661832	ic				
**166**	Er	pNFkBp65 (S529)	K10-895.12.50	Fluidigm	3166006A	AB_2847867	ic				
**167**	Er	CD38	HIT2	Biolegend	303502	AB_314354	sf	x		x	x
**168**	Er	CD16	3G8	Biolegend	302002	AB_314202	sf	x	x		x
**169**	Tm	CD25	MA-251	Biolegend	356102	AB_2561752	sf				x

**170**	Er	Siglec1	7-239	Miltenyi Biotec	130-108-017	AB_2655556	sf				
**171**	Yb	ZAP70/Syk (Y319/352)	17a	Fluidigm	3171005A	AB_2827884	ic				
**172**	Yb	pS6 (S235/S236)	N7-548	Fluidigm	3172008A		ic				
**173**	Yb	IgD	IA6-2	Biolegend	348202	AB_10550095	sf			x	
**174**	Yb	HLA-DR	L243	DRFZ	n.a.		sf	x	x	x	x
**176**	Yb	CD56	REA196	Miltenyi	130-108-016	AB_2658728	sf	x			x
**191**	Ir	DNA intercalator	n.a.	Fluidigm	201192A	n.a.	ic				
**193**	Ir	DNA intercalator	n.a.	Fluidigm	201192A	n.a.	ic				
**195**	Pt	CD3	UCHT1	DRFZ	n.a.		sf	x			x
**196**	Pt	CD8	GN11	DRFZ	n.a.		sf	x			x
**198**	Pt	CD45	Hi30	Biolegend	304002	AB_314390	sf	x	x	x	x
**209**	Bi	CD11b	ICRF44	Biolegend	301302	AB_314154	sf				
**n.a. - not applicable**											

Table ST2: Clinical characteristics of SLE patients

**sample ID**	**sex**	**prednisone**	**hydroxychloro-quine**	**MTX**	**azathioprin**	**belimumab**	**mycophenolate mofetil**	**SLEDAI-2K**	**complement use**	**mucocutaneous**	**musculoskeletal**	**cardio-respiratory**	**renal involvement**	**vasculitis**	**neuropsychia-tric**	**C3 [mg/L]**	**C4 [mg/L]**
**SLE_001**	f	1	0	0	0	0	0	12	0	1	1	1	1	0	0	910	70

**SLE_002**	m	1	1	1	0	1	0	10	1	1	1	0	0	0	0	820	180
**SLE_003**	f	1	1	0	0	0	0	10	1	1	1	0	0	0	0	630	60
**SLE_004**	f	1	0	0	0	0	0	10	1	1	1	0	0	0	0	660	60
**SLE_006**	f	1	1	0	0	0	0	4	1	0	0	0	0	0	0	590	110
**SLE_007**	f	1	1	0	0	0	0	8	1	0	1	0	0	0	0	820	90
**SLE_008**	f	1	1	1	0	0	0	8	1	1	0	0	0	0	0	950	80
**SLE_009**	m	1	0	0	0	0	0	16	1	0	0	0	1	1	0	370	40
**SLE_010**	f	0	1	0	1	0	0	8	1	0	0	0	1	0	0	600	80
**SLE_011**	f	1	1	0	0	0	0	8	1	1	0	0	0	0	0	730	80
**SLE_012**	m	1	0	0	0	0	0	12	1	0	0	0	1	0	0	470	90
**SLE_014**	f	0	0	0	0	0	0	4	1	1	1	0	0	0	1	870	180
**SLE_015**	f	0	1	0	0	0	0	5	1	0	0	0	0	0	0	970	50
**SLE_016**	f	1	1	0	1	0	0	6	1	1	0	0	0	0	0	620	40
**SLE_017**	f	1	1	0	1	1	0	8	1	1	1	0	0	0	0	640	100
**SLE_018**	f	1	1	0	1	0	0	14	0	1	0	0	0	1	0	990	150
**SLE_019**	f	1	1	1	0	1	0	10	1	1	0	1	0	1	1	840	170

**SLE_020**	f	1	0	0	0	0	1	10	1	0	0	0	1	0	0		
**SLE_021**	f	0	1	0	0	0	0	4	0	0	1	0	0	0	0	1260	350
**SLE_022**	f	0	1	0	0	0	0	2	0	1	0	0	0	0	0	1140	160

MTX = methotrexate, SLEDAI-2K = SLE disease activity index 2000, C3 = C3 complement protein, C4 = C4 complement protein, 0 = no, 1 = yes

Table ST3: SIGLEC-1 levels and autoantibody profiles of SLE patients.

**sample ID**	**SIGLEC-1 [molecules/cell]**	**RF IgA**	**RF IgM**	**ACPA**	**Ro**	**La**	**U1RNP**	**RNP70**	**Sm**	**Scl70**	**Jo1**	**CENP**	**dsDNA**	**nucleo-somes**
**SLE_001**	19746	1	1	1	1	0	0	0	0	0	0	0	406	1
**SLE_002**	7533	0	0	0	1	1	0	0	1	0	0	0	20	1
**SLE_003**	16794	0	0	1	1	0	0	0	0	0	0	0	109	1
**SLE_004**	7638	1	1	1	0	0	0	1	0	0	0	0	103	1
**SLE_006**	7236	0	0	0	0	0	0	0	0	0	0	1	57.9	1
**SLE_007**	3144	0	0	0	0	0	0	0	0	0	0	0	>200	1

**SLE_008**	6986	1	1	1	1	1	0	0	0	0	0	0	109	1
**SLE_009**	11293	0	0	0	1	0	1	1	0	0	0	0	>200	1
**SLE_010**	7996	0	0	0	0	0	0	0	0	0	0	0	>200	1
**SLE_011**	1241	0	0	0	1	0	0	0	0	0	0	0	38.1	1
**SLE_012**	18382	1	1	1	1	0	0	0	0	0	0	0	>200	1
**SLE_014**	9390	1	0	0	1	1	0	0	0	0	0	0	10	0
**SLE_015**	1475	0	0	0	1	1	0	0	0	0	0	0	>200	1
**SLE_016**	8069	1	1	1	1	1	1	1	1	0	0	0	40.5	1
**SLE_017**	13961	0	0	0	0	0	0	0	0	0	0	0	>200	1
**SLE_018**	1909	0	0	0	0	0	0	0	0	0	0	0	41.2	1
**SLE_019**	18442	0	0	0	1	0	0	0	0	0	0	0	63	1
**SLE_020**	7996	0	0	0	1	0	1	1	1	0	0	0	69	1
**SLE_021**	1266	0	0	0	0	0	0	0	0	0	0	0	8.0	1
**SLE_022**	11443	0	0	0	1	0	0	0	0	0	0	0	10.4	0

SIGLEC-1 = sialic acid binding Ig-like lectin-1, RF = rheumatoid factor, IgM/A = immunoglobulin M/A, ACPA = anti-citrullinated protein antibody, Ro = anti-SSA/Ro, La= anti-SSB/La, RNP = ribonucleoprotein, Sm = Smith, Scl70 = anti-topoisomerase I, J01= anti-histidyl-tRNA-synthetase, CENP = centromeric protein, dsDNA = double-stranded DNA, 0 = no, 1 = yes
